# The petrosal and bony labyrinth of extinct horses (Perissodactyla, Equidae) and their implications for perissodactyl evolution

**DOI:** 10.7717/peerj.20484

**Published:** 2026-01-05

**Authors:** Owen Axel Goodchild, Sydney Nicole Rosen, Bastien Mennecart, Jin Meng, Jérémy Tissier

**Affiliations:** 1 Richard Gilder Graduate School, American Museum of Natural History, New York, New York, United States; 2 Department of Biological Sciences, Vanderbilt University, Nashville, Tennessee, United States; 3 Naturhistorisches Museum Basel, Basel, Switzerland; 4 Department of Vertebrate Paleontology, American Museum of Natural History, New York, New York, United States; 5 Palaeobiosphere Evolution Unit, Royal Belgian Institute of Natural Sciences, Brussells, Belgium

**Keywords:** Perissodactyla, Equidae, Petrosal, Bony labyrinth, Phylogeny, Morphology, Paleontology

## Abstract

Perissodactyla, or odd-toed ungulates, are represented today by 16 species of rhinoceroses, tapirs, and horses. Perissodactyls were much more diverse in the past, having a rich fossil record spanning from the earliest Eocene (~56 Ma) to recent, including a myriad of extinct lineages. Despite over a century of study, the inter-relationships of some extinct perissodactyl families remain poorly resolved. New morphological characters are needed to help solve this issue. Recent studies suggest that the ear region, *i.e*., the petrosal and the bony labyrinth of the inner ear, is a valuable source of morphological characters for mammalian phylogenetic analyses. The petrosal is the bony structure protecting the inner ear, the organs of hearing and balance in mammals. However, perissodactyl petrosals are poorly documented and have not been used in such a phylogenetic framework. In this study, we describe the petrosals and inner ears of five European fossil equid taxa and perform a preliminary phylogenetic analysis. Despite its small sample size, our phylogenetic analysis recovers important groupings, which suggests the petrosal is phylogenetically informative in equids. This study supports the relevance of the ear region for phylogenetic inference and its potential to better resolve long-contentious relationships within Perissodactyla.

## Introduction

Today, Perissodactyla Owen, 1848, also known as odd-toed ungulates, are represented by 16 living species of rhinoceroses (n = 5), tapirs (n = 4), and horses (n = 7). Perissodactyls have a rich fossil history extending to the early Eocene ~56 million years ago (Ma; Bai, Wang & Meng, 2018). In addition to the ancestors of living perissodactyl groups, the perissodactyl fossil record contains several extinct families like the clawed Chalicotheriidae and bony-horned Brontotheriidae (Bai, Wang & Meng, 2018). Despite over a century of study, the interrelationships between extinct perissodactyl families and the relationships within those families remain controversial. Phylogenetic analyses using craniodental characters have longstanding issues, such as the internal relationships of Rhinocerotoidea (Tissier et al., 2018; Bai et al., 2020).

The discrepancies among these recent phylogenies highlight the necessity to investigate other structures of perissodactyl anatomy for new phylogenetically relevant characters. The petrosal is the paired basicranial bone housing the inner ear, which comprises the organs of balance (semicircular canals) and hearing (cochleae) in mammals (O’Leary, 2010). The bony labyrinth is the bony cavity within the petrosal bone, which houses the semicircular canals and cochlea, and is thus often considered as a good representation of the morphology of the inner ear. In many mammals other than perissodactyls, the petrosal and bony labyrinth are increasingly used in phylogenetic analyses and in understanding the paleobiology of these animals (Mennecart & Costeur, 2016; Mennecart et al., 2016; Aguirre-Fernández et al., 2017; Costeur et al., 2017, 2018a, 2018b; Aiglstorfer et al., 2017; Benoit et al., 2020; Mennecart et al., 2020; Evin et al., 2022; Wang et al., 2022; Mennecart et al., 2022; Orliac et al., 2023; Zhang & Tong, 2024). The petrosal and bony labyrinth have historically been a challenge to study because the bony labyrinth is completely enclosed in the petrosal, while the petrosal is often enclosed in the skull. Computed tomography (CT) allows for the visualization of internal and external details of the petrosal, and the generation of endocasts of the bony labyrinth within. The petrosal of perissodactyls is relatively poorly known and has yet to be used in large-scale phylogenetic analyses (see Mateus, 2018 for a review).

This study aims to describe the petrosal, bony labyrinth, and stapes (when preserved) of five different extinct equids, explore their morphological variation, and assess whether the petrosal characters presented by O’Leary (2010) and Mateus (2018) are phylogenetically informative for these taxa.

## Materials and Methods

### Taxonomy and specimens

**Figure 1:**
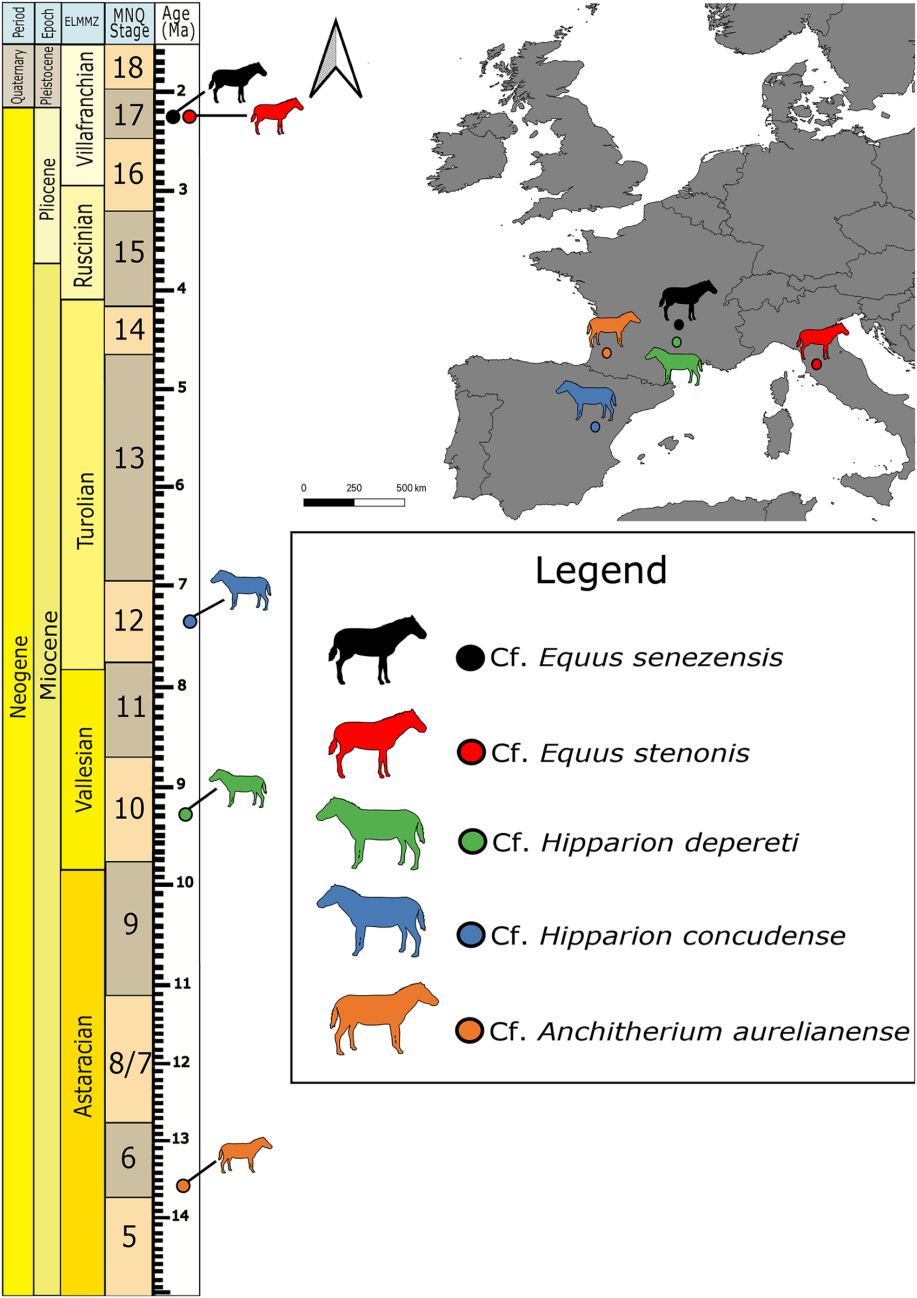
Map of fossil sites for equid specimens in this study (right) and the corresponding stratigraphic and biostratigraphic ages of those sites (left). ELMMZ, European Large Mammal Mega-Zone; MNQ, Mammal Neogene/Quaternary Biostratigraphic Stage. Image Source Credit: Zimices, https://www.phylopic.org/images/0df28002-4d73-4679-95c0-7f271e4c89c1/equus-stenonis, CC BY-SA 4.0.

This study involves seven previously-identified petrosal specimens (Table 1; Fig. 1) from five European fossil equids housed in the collections of the Natural History Museum of Basel, Switzerland (NMB) that were previously identified in the collection. We confirmed the identification based on the reported taxonomic diversity from each locality, and by comparison with the petrosal of extant *Equus* (*e.g*., in O’Leary, 2010 and Danilo et al., 2015), as well as with Artiodactyla (O’Leary, 2010), *Tapirus* (O’Leary, 2010; Mateus, 2018) and *Ceratotherium* (Robert et al., 2021). We also had access to the petrosals of *Equus caballus* (AMNH FM 118) and *Tapirus terrestris* (AMNH FM 14103) described by O’Leary (2010) for comparison and phylogenetic analysis.

**Table 1:** Petrosal specimens examined in the study.

Specimen and taxon	Locality	Age	Left or right	Bony labyrinth	Stapes	Image dimensions	Inter-slice spacing (mm)
NMB.San.15063 cf. *Anchitherium aurelianense*	Sansan, Gers, France	Astaracian (MN 6)	Right	No (no contrast)	No	397 × 519 × 311	0.055 × 0.055 × 0.055
NMB.A.Mo.655 cf. *Hipparion depereti*	Montredon, Occitanie, France	Vallesian (MN 10)	Left	Yes	No	672 × 729 × 600	0.056249 × 0.056249 × 0.056250
NMB.Ccd.3 cf. *Hipparion concudense*	Concud 3, Teruel, Spain	Turolian (MN 12)	Left	Yes	Yes	410 × 656 × 530	0.056249 × 0.056249 × 0.056250
NMB.V.A.2753 cf. *Equus stenonis*	Valdarno, Tuscany, Italy	Villafranchian (MN 17)	Left	Yes	Yes	865 × 877 × 751	0.05625 × 0.05625 × 0.05625
NMB.Se.141 cf. *Equus senezensis*	Senèze, Alpes-de-Haute-Provence, France	Villafranchian (MN 17)	Left	Yes	Yes	594 × 707 × 623	0.056249 × 0.056249 × 0.056250
NMB.Se.141 cf. *Equus senezensis*	Senèze, Alpes-de-Haute-Provence, France	Villafranchian (MN 17)	Right	Yes	No	583 × 893 × 561	0.056249 × 0.056249 × 0.056250
NMB.Se.554 cf. *Equus senezensis*	Senèze, Alpes-de-Haute-Provence, France	Villafranchian (MN 17)	Right	Yes	No	714 × 694 × 699	0.055 × 0.055 × 0.055

Cf. *Anchitherium aurelianense* Cuvier, 1825 is the oldest equid in our study (Agustí & Antón, 2002). The petrosal imaged here comes from the famous Middle Miocene locality of Sansan (France) dated to the Astaracian European Land Mammal Age ~15 Ma (Alberdi, Ginsburg & Rodríguez, 2004). Its identification in our study remains tentative, as we could not directly compare it with another clearly identified specimen. *Hipparion* belongs to a large group of fossil equids, the Hipparionini, from across North America, Asia, Europe, and Africa (Bernor et al., 2021). The two *Hipparion* specimens in this study come from two different sites: Montredon (Vallesian, 11-9 Ma; France) and Concud (upper Turolian, ~5 Ma, Spain; Forstén, 1982). All the *Equus* material in our sample comes from the Early Pleistocene (Villafranchian), one from Valdarno (Italy) and three from Senèze (France). They belong to the stenonine lineage that consists of Early Pleistocene European and African *Equus* (Cirilli et al., 2021a). The specimen from Valdarno belongs to cf. *E. stenonis*, while the specimens from Senèze belong to cf. *E. senezensis* (Cirilli et al., 2021b).

### CT scans and segmentation

The equid material for this project was scanned at the Biomaterials Science Centre of the University of Basel, Switzerland, using a Phoenix Nanotom, GE. Tomograms were segmented using 3D Slicer (Fedorov et al., 2012) to extract the petrosal, the digital endocast of the bony labyrinth, and the stapes. 3D models representing seven petrosal bones, six bony labyrinths, and three stapes were generated in 3D Slicer. All tomograms and 3D models of petrosals, bony labyrinths, and stapes are available for download on Morphosource (https://www.morphosource.org/projects/000720375?locale=en).

### Measurements

We made point to point digital measurements using the “Measuring Tool” in MeshLab2022.02 (Cignoni et al., 2008). We followed the linear measurement methods outlined in Ekdale (2013: figure 3). We measured the height and width of the cochlea used for the calculation of the aspect ratio and the height, width, and length of the semicircular canals used for the calculation of the radius of curvature (Table 2). We measured the angles between the semicircular canals in 3D Slicer using the “create new angle” tool.

**Table 2:** Bony labyrinth measurements of fossil equids.

Specimen no.	Taxon ID	Source	ASC H/W aspect ratio	PSC H/W aspect ratio	LSC H/W aspect ratio	Cochlear turns	Cochlea aspect ratio	Angle ASC-LSC	Angle ASC-PSC	Angle LSC-PSC
AMNH FM 55267	*Xenicohippus osborni*	Ravel & Orliac (2015)	1.00	0.98	1.13	_	_	–	–	–
NMB.A.Mo.655	cf. *Hipparion depereti*	This study	1.13	1.25	0.83	2.5	0.55	93.1	82.2	99.6
NMB.Ccd.3	cf. *Hipparion concudense*	This study	0.99	1.12	0.97	2.5	0.5	80.7	103.3	85.8
NMB.V.A.2753	cf. *Equus stenonis*	This study	1.17	1.13	1.01	2.5	0.56	98.6	91.8	93.6
NMB-Se-554	cf. *Equus senezensis*	This study	0.87	0.89	0.97	2.5	0.44	87.0	88.1	84.5
NMB-Se-141 (left)	cf. *Equus senezensis*	This study	1.05	1.09	1.05	2.5	0.58	83.3	92.1	95.8
NMB-Se-141 (right)	cf. *Equus senezensis*	This study	1.01	0.98	1.12	2.5	0.51	87.3	85.9	88.5
TMM-M-171	*Equus caballus*	Ekdale (2013)	0.93	1.15	1.04	2.5	0.41	84.7	93.3	90.1

**Note:**

ASC, anterior semicircular canal; LSC, lateral semicircular canal; PSC, Posterior semicircular canal.

### Character scores and phylogenetic analysis

We constructed a character matrix in Mesquite, combining characters from the petrosal and bony labyrinth, which is provided in Nexus format in File S1. The list of characters and characters states is included in the matrix file, and detailed in File S2. We scored the 3D models of the petrosal and stapes with characters from Spaulding, O’Leary & Gatesy (2009; available in morphobank: http://dx.doi.org/10.7934/X188). We retained 33 characters which exclusively concern the petrosal bone, and the two characters of the stapes (characters 65 and 66 of Spaulding, O’Leary & Gatesy (2009)). We excluded characters from the auditory bulla, which was not preserved in our fossil specimens. We added nine characters from Mateus (2018) for a total of 42 petrosal characters (see File S1). We scored the 3D models of the bony labyrinth according to the 6 discrete characters of Ekdale (2013; available in morphobank: http://dx.doi.org/10.7934/X1905). We scored *Equus przewalskii* in the matrix based on the descriptions and figures of Danilo et al. (2015) and used the original scores of *Hyopsodus*, *Tapirus terrestris*, and *Equus caballus* from Spaulding, O’Leary & Gatesy (2009) as well as those of *Equus* from the matrix of Ekdale (2013) and of *Hyopsodus* and *Tapirus terrestris* from Mateus (2018). In total, our matrix includes 50 characters (nine are parsimony informative in our analysis and 29 are constant) and 10 terminal taxa.

We performed a maximum parsimony (MP) analysis using PAUP4 (Swofford, 2002). Given the small number of taxa in our sample, we used the exhaustive search function to search all possible tree topologies to obtain the most parsimonious tree(s). *Hyopsodus* was set as the outgroup, and all characters are considered unordered. The optimization settings were set on ACCTRAN.

We performed a Bootstrap analysis in PAUP4, using 100 replicates with a full heuristic search algorithm. For each boostrap replication, 1,000 heuristic replicates were done, holding 100 trees at each step, with a TBR algorithm and a reconnection limit of 8.

### Anatomical terminology

The anatomical terminology used for the description of the stapes follows Orliac & Billet (2016). The terminology used for the petrosal follows O’Leary (2010), and the terminology of the inner ear follows Ekdale (2013). More specifically, we follow the terminology of Robson & Theodor (2025) for the subarcuate depression of the petrosal (rather than fossa).

### Biostratigraphy

The stratigraphical framework is based on the geological timescales and European Land Mammal Ages (ELMA) for the Neogene (Raffi et al., 2020).

## Results

### Systematic Paleontology

Mammalia, Linnaeus, 1758

Perissodactyla, Owen, 1848

Equidae, Gray, 1821

Anchitheriinae, Leidy, 1869

cf. *Anchitherium*, Von Meyer, 1844

cf. *Anchitherium aurelianense*, Cuvier, 1825

**Material**

An isolated right petrosal, NMB.San.15063

**Locality and age**

Sansan, Gers, France; Miocene, Astaracian (MN 6)

**Figure 2:**
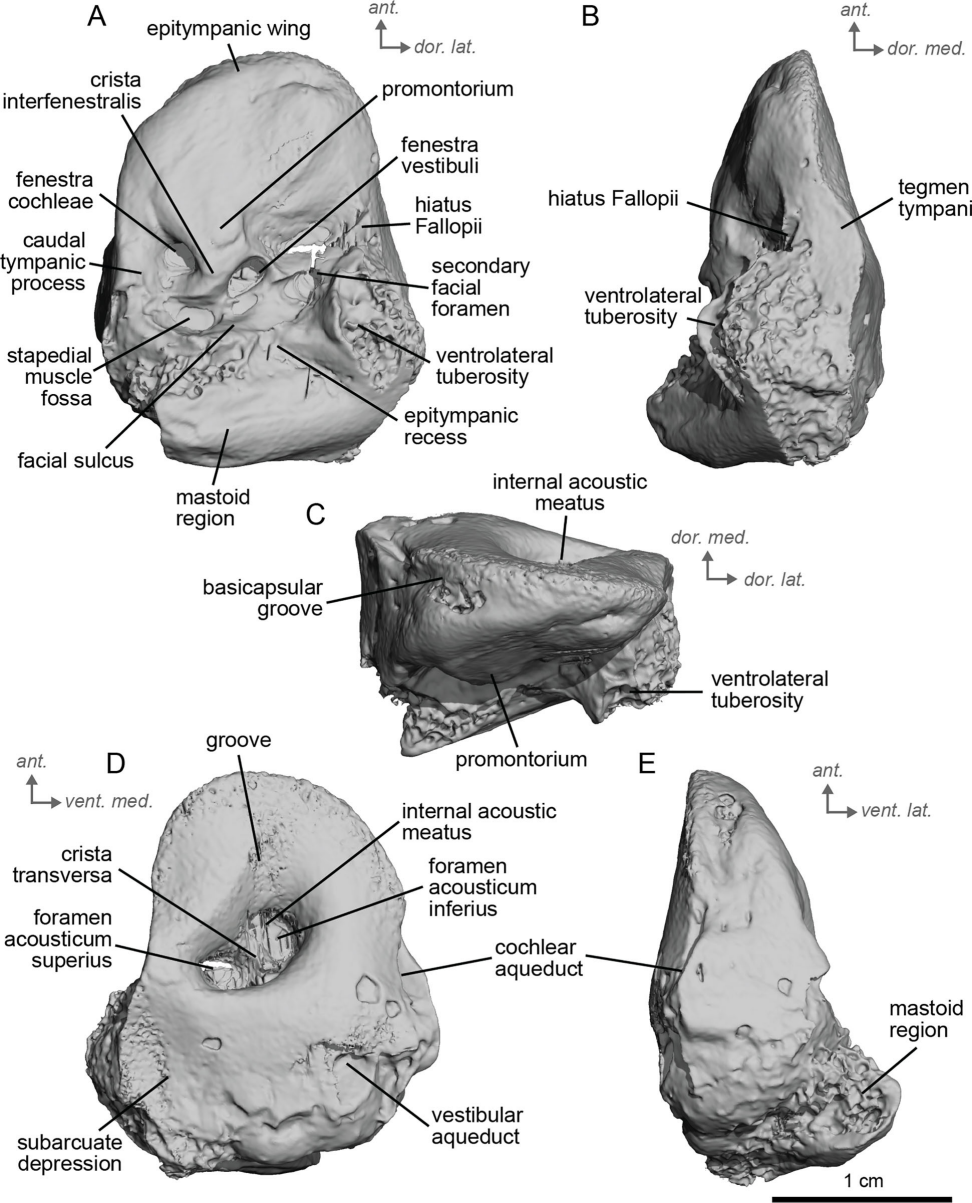
Right petrosal (NMB.San.15063) of cf. *Anchitherium aurelianense* from Sansan (mirrored). (A) Ventrolateral view. (B) Ventromedial view. (C) Anterior view. (D) dorsomedial view. (E) Dorsolateral view. *Ant* = anterior, *dor.*, = dorsal, *lat*. = lateral, *med*. = medial, *vent*. = ventral.

**Description and comparison.** The petrosal of cf. *Anchitherium aurelianense* (NMB.San.15063; Fig. 2) is 2.11 cm long anteroposteriorly. The specimen is largely complete, with minor damage to the mastoid region. The bony labyrinth could not be segmented in this specimen due to the absence of contrast between the sediment infilling the bony labyrinth and the petrosal bone.

The petrosal of cf. *A. aurelianense* is markedly different in several aspects from that of *Equus caballus* (O’Leary, 2010). In anterior view (Fig. 2C), cf. *A. aurelianense* lacks the endocranial projection of the superiormost aspect of the petrosal seen in *Equus*. The *crista interfenestralis* is broader and more rounded than in *E. caballus*. The epitympanic wing is small, forming a low protrusion from the promontorium. The wing is rounded rather than pointed and does not protrude. The subarcuate depression is very shallow. The *hiatus Fallopii* is small and opens on the ventromedial border of the petrosal, close to the *tegmen tympani* (Fig. 2B). The stapedial muscle fossa is oval-shaped and located in the facial *sulcus*, below the *crista interfenestralis* separating the *fenestrae vestibuli* and *cochleae*. The *fenestra cochleae* is round, while the *fenestra vestibuli* is oval. Cf. *Anchitherium* possesses a notably smaller *tegmen tympani* than *E. caballus* (O’Leary, 2010). Unlike in *E. caballus*, the *tegmen tympani* is flattened and is not prominent in dorsolateral view (Fig. 2B). The surface of the *tegmen tympani* is smooth, forming an angled surface anteromedial to the mastoid region. The *tegmen tympani* lacks raised bumps and the *hiatus Fallopii* excavates a portion of its medial edge.

The dorsomedial surface of the petrosal in cf. *A. aurelianense* is largely smooth, like *E. caballus* (O’Leary, 2010), but with some rugosity along the anterior margin and along the floor of the groove anterior to the internal acoustic meatus (Fig. 2D). Since this petrosal was found as an isolated element we interpret this rugosity as the result of taphonomic weathering. The *crista transversa* is a sinuous ridge of bone between the superior and inferior acoustic foramina, as in *E. caballus*, but is thicker. The cochlear aqueduct is small and slit-like (Figs. 2D, 2E). The vestibular aqueduct forms a round, open hole (Fig. 2D).

The peculiar nature of the *tegmen tympani* in the petrosal of cf. *Anchitherium* recalls the “uninflated” condition seen in the early diverging eutherian *Protungulatum* (O’Leary, 2010). As in *Protungulatum*, the *tegmen tympani* is flat in cf. *Anchitherium*. The surface is moderately raised in dorsomedial view relative to the internal acoustic meatus, while in *Protungulatum* the *tegmen tympani* is flatter in dorsomedial view. The *tegmen tympani* morphology of cf. *Anchitherium* is somewhat intermediate between the uninflated *tegmen tympani* of *Protungulatum* and the smaller but inflated *tegmen tympani* of *Hipparion*. It is puzzling, then, that the literature reports the earlier diverging equid *Orohippus* as having an inflated *tegmen tympani* (Cifelli, 1982). Indeed, all the fossil tapirs (Mateus, 2018) referred to have an inflated *tegmen tympani*. Cf. *A. aurelianense* lacks an anterior process of the *tegmen tympani*. A ventrolateral tuberosity was present in cf. *A. aurelianense* (Figs. 2A–2C). Medial to the external acoustic meatus is a relatively deep epitympanic recess (Fig. 2A). Cf. *A. aurelianense* lacks a distinct stylomastoid notch.

The petrosal is narrow in ventromedial view (Fig. 2E), widening into a fan-shaped mastoid region like in *E. caballus* (O’Leary, 2010). The mastoid region is very incomplete. It was considered here to be large as per the definition of O’Leary (2010), but is notably smaller than that of *E. caballus*. The preserved element of the mastoid is consistent with a wedge shape as described in O’Leary (2010).

Equinae, Steinmann & Döderlein, 1890

Hipparionini, Quinn, 1955

*Hipparion*, Christol, 1832

cf. *Hipparion depereti*, Sondaar, 1974

**Material**

An isolated left petrosal, NMB.A.Mo.655

**Locality and age**

Montredon, France; Late Miocene, Vallesian (MN 10).

**Figure 3:**
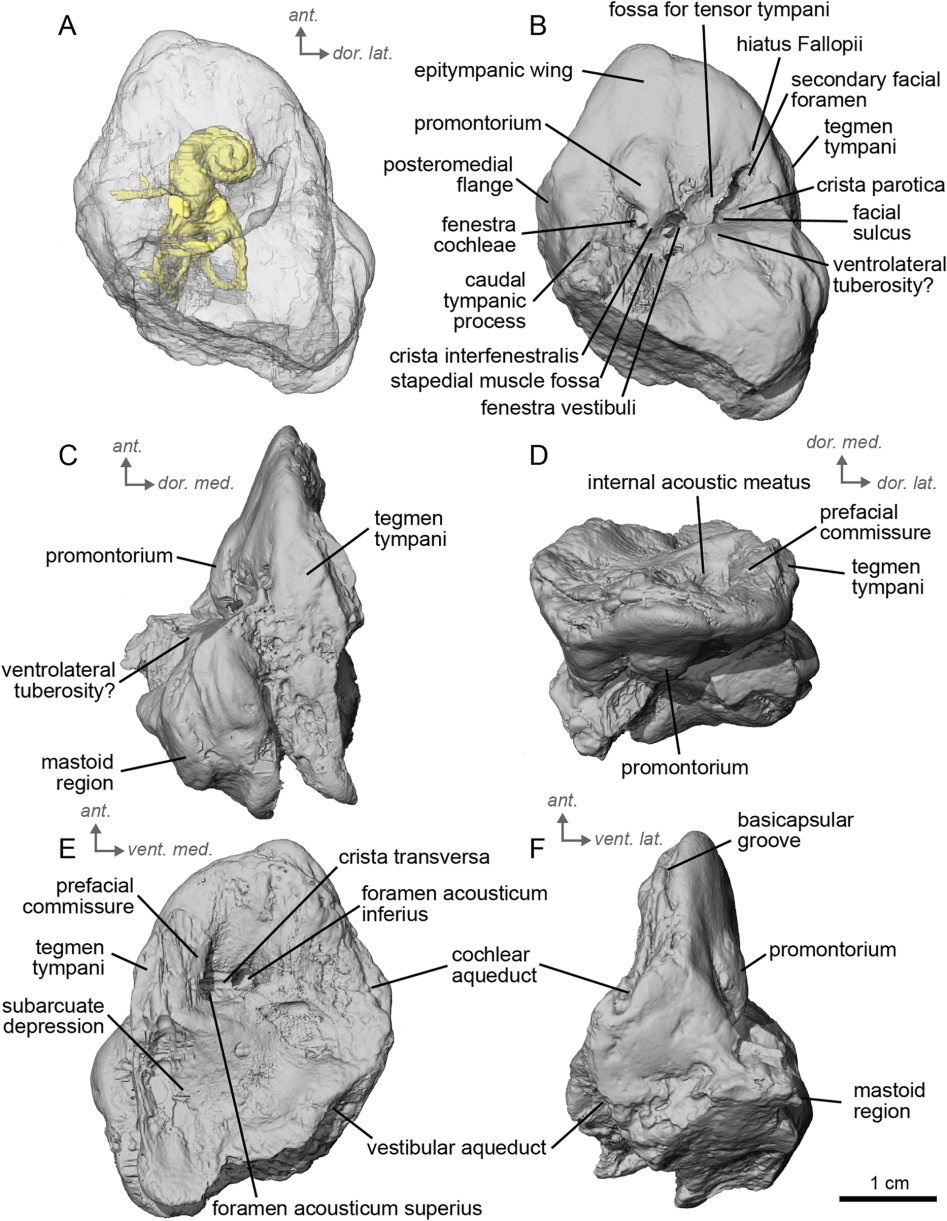
Left petrosal (NMB.A.Mo.655) of cf. *Hipparion depereti* from Montredon. (A) Ventrolateral transparent view. (B) Ventrolateral opaque view. (C) dorsolateral view. (D) Anterior view. (E) Dorsomedial view. (F) Ventromedial view. *Ant* = anterior, *dor*. = dorsal, *lat*. = lateral, *med*. = medial, *vent*. = ventral.

**Description and comparison.** The Montredon *Hipparion*, cf. *H. depereti*, specimen NMB.A.Mo.655, is a largely complete isolated petrosal measuring 3.9 cm in length, with minor damage to the *tegmen tympani* and the mastoid region. Segmentation of its bony labyrinth was made challenging by the presence of very dense infilling (possibly iron). This petrosal is intermediate between cf. *Anchitherium* and *Equus*. Notably, the petrosal of cf. *H. depereti* is more massively constructed and broader than that of cf. *A. aurelianense*. There are notable distinctions from *Equus*, however. The *promontorium* gives rise to the epitympanic wing (Fig. 3B), which is small and rounded rather than pointed as in *E. caballus* (O’Leary, 2010), but longer than in cf. *Anchitherium*.

Between the epitympanic wing and the *tegmen tympani* lies the opening for the *hiatus Fallopii* (Fig. 3B), which is very small. The fossa for the *muscularis tensor tympani* is located between the *hiatus Fallopii* and the *fenestra vestibuli*. The *fenestra cochleae* is round, and the *fenestra vestibuli* is oval. The stapedial muscle fossa is deep and round in the facial sulcus, just below the *crista interfenestralis*. The stylomastoid notch is thin and rather shallow. The *tegmen tympani* (Figs. 3C, 3D) is more greatly inflated than in cf. *A. aurelianense* but much less than in *E. caballus*. There is no anterior process of the *tegmen tympani*. In dorsomedial view (Fig. 3E), the surface of the area around the internal acoustic meatus is smooth. However, this specimen has been abraded, making the surface appear more rugose than in life. The subarcuate depression is wide and shallow, as in *E. caballus* (O’Leary, 2010) and the petromastoid canal is absent. In dorsolateral view (Fig. 3C), the petrosal is narrow anteriorly and expands into a fan-shaped mastoid region posteriorly. The medial surface of the petrosal is flat. The basicapsular groove can be seen along the dorsomedial margin of the petrosal (Fig. 3F). The cochlear aqueduct is a very small hole at the ventromedial margin (Figs. 3E, 3F), although located more ventrally than in *Tapirus terrestris* (like *E. caballus*). The vestibular aqueduct is visible but the mastoid part of the petrosal is broken. The ventral knob-like area of the mastoid is well-preserved, but lacking the dorsal point seen in *E. caballus* (O’Leary, 2010).

**Figure 4:**
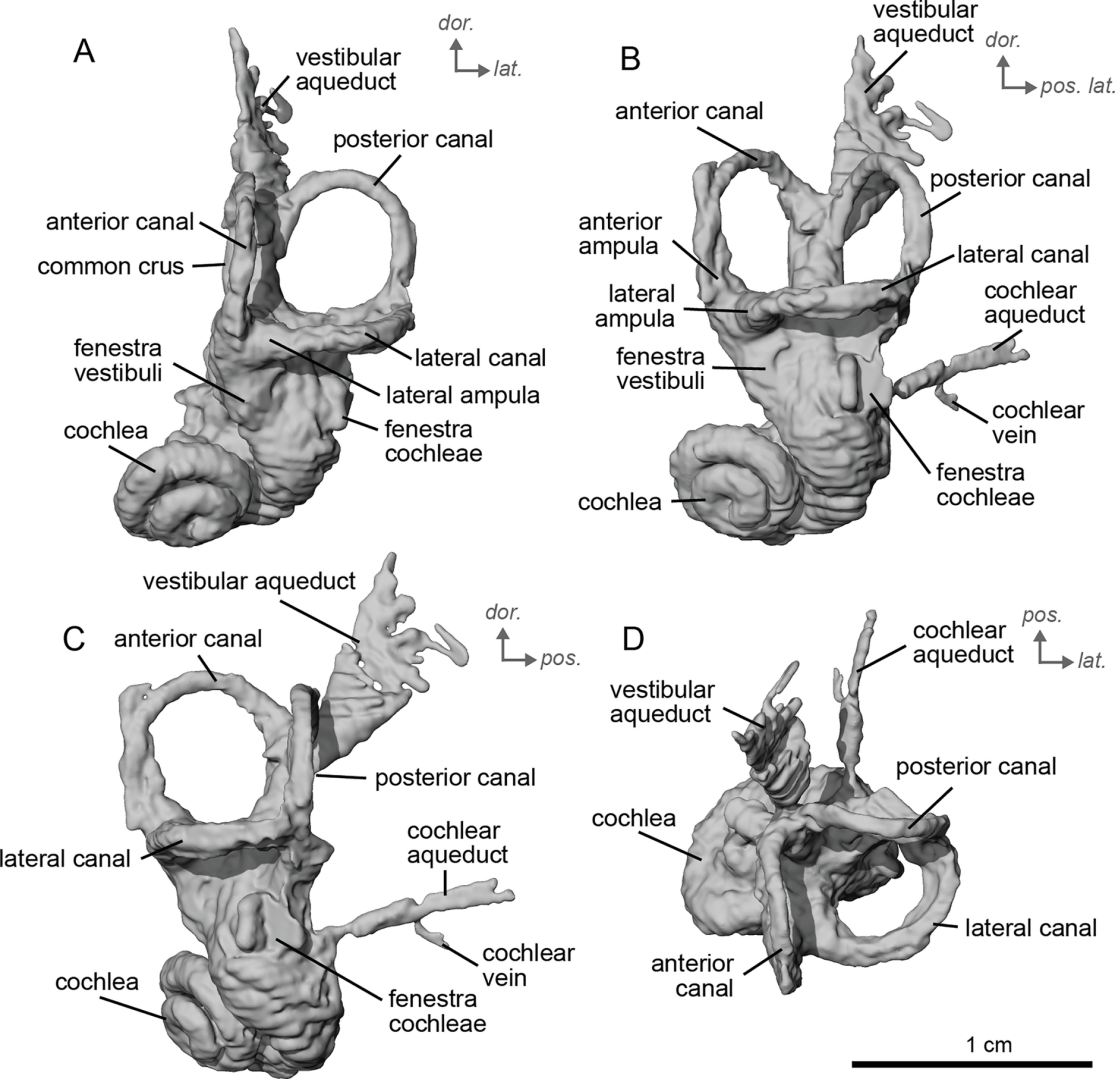
Endocast of the left bony labyrinth of cf. *Hipparion depereti* (NMB.A.Mo.655) from Montredon. (A) Anterior view. (B) Posterior view. (C) Lateral view. (D) Dorsal view. *Dor*. = dorsal, *lat*. = lateral, *pos*. = posterior.

The bony labyrinth of NMB.A.Mo.655 (Table 2) is more poorly preserved than the other specimens in this study, as dense infilling obscured its shape. Nevertheless, the gross morphology of the bony labyrinth can be described and discussed. It shows little post-mortem deformation. The bony labyrinth fills much of the volume of the petrosal, but not to the extent observed in *Hyopsodus lepidus* (Ravel & Orliac, 2015). The presence of a secondary bony lamina is unclear due to poor preservation or a genuine absence. In the bony labyrinth of the earlier diverging equid *Xenicohippus osborni*, no secondary bony lamina was observed, although deformation made that observation questionable (Ravel & Orliac, 2015). In the more derived *E. caballus*, the secondary bony lamina is weakly developed (Ekdale, 2013). The cochlear spiral aspect ratio (0.55) is higher than that of *E. caballus*, although notably lower than that observed in *Hy. lepidus* (Ravel & Orliac, 2015). The cochlea completes two and a half turns and is loosely coiled (Fig. 4B) like *E. caballus* (Ekdale, 2013). As in *X. osborni*, the cochlea is elliptical, with the anteroposterior axis longer than the mediolateral axis. The cochlear aqueduct is straight, narrowing as it nears its external aperture as in *E. caballus* (Ekdale, 2013), and short. The lateral semicircular canal widens anteriorly to form a lateral ampulla (Fig. 4A). Posteriorly, the lateral semicircular canal and posterior semicircular canal appear to form a secondary common crus (Fig. 4), unlike *E. caballus*, although this may be an artefact of poor resolution in the model (Ekdale, 2013). Like both *X. osborni* and *E. caballus*, the arc of the anterior semicircular canal possesses the largest radius and the greatest height of the three (Ravel & Orliac, 2015). The lateral semicircular canal attaches more dorsally to the vestibule than the posterior semicircular canal does. The angles between the posterior and lateral semicircular canals, and between the anterior and lateral semicircular canals, lie at nearly a right angle to one another (93.1°) (Fig. 4C). The angle between the posterior and anterior canals is more acute (82.2°) (Fig. 4D). The anterior and posterior canals are relatively rounded, while the lateral one is ovoid in shape. The long endolymphatic sac is triangular in shape and posteriorly projected. It starts high, almost at the dorsal end of the common crus, but this could also be due to the poor preservation of the specimen. There is no clear distinction between the fenestrae vestibuli and cochleae due to the preservation of the specimen. The cochlea is not in contact with the vestibule.

cf. *Hipparion concudense*, Pirlot, 1956

**Material**

An isolated left petrosal, NMB.Ccd.3

**Locality and age**

Concud 3, Teruel, Spain; Late Miocene, Turolian (MN 12).

**Figure 5:**
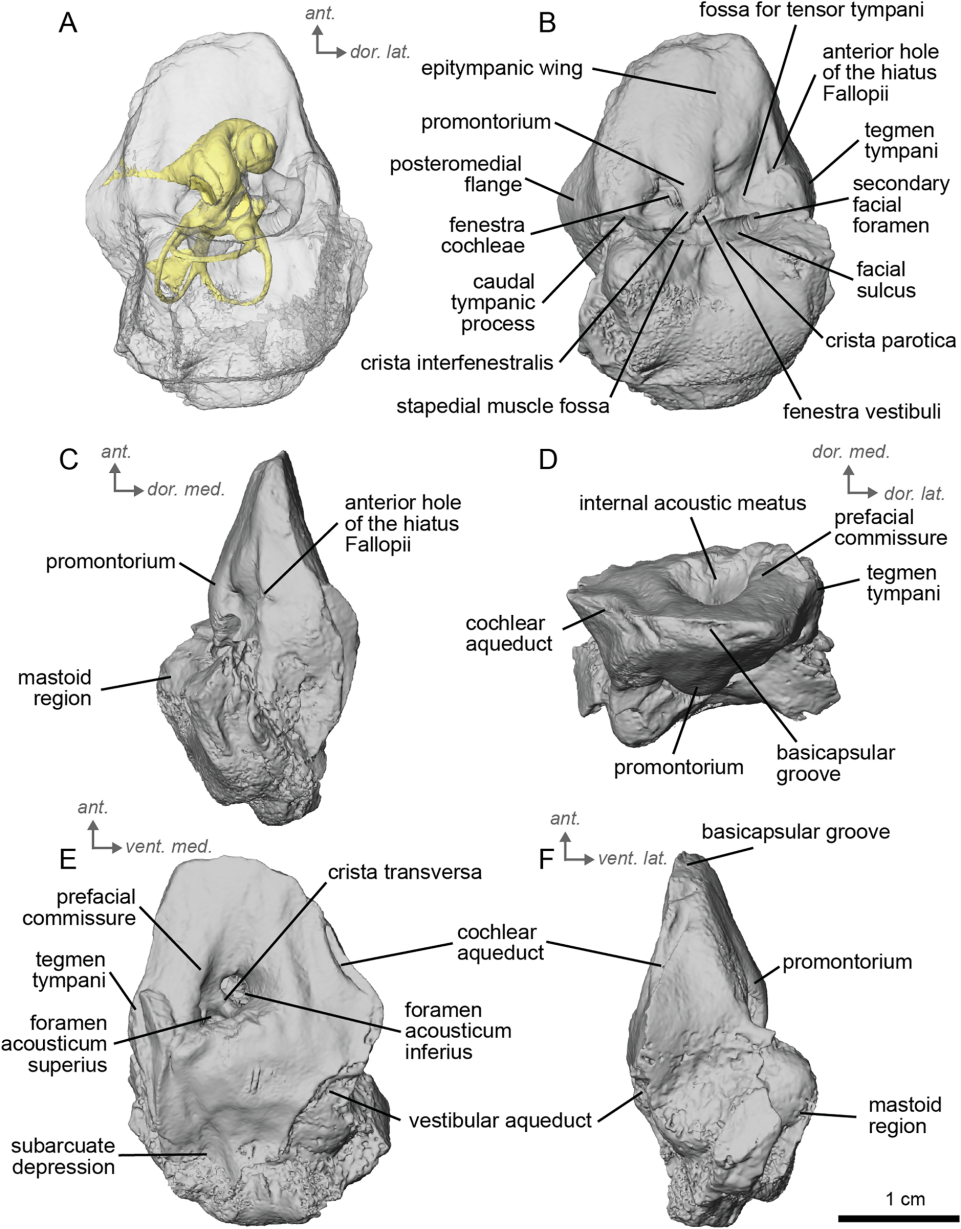
Left petrosal (NMB.Ccd.3) of cf. *Hipparion concudense* from Concud 3. (A) Ventrolateral transparent view. (B) Ventrolateral opaque view. (C) dorsolateral view. (D) Anterior view. (E) Dorsomedial view. (F) Ventromedial view. *Ant* = anterior, *dor*. = dorsal, *lat*. = lateral, *med*. = medial, *vent*. = ventral.

**Description and comparison.** The Concud 3 *Hipparion*, cf. *H. concudense*, specimen is an almost entirely complete petrosal, with only minor damage to the mastoid region, measuring 3.15 cm in length. It preserved a stapes fallen within the bony labyrinth. The petrosal of cf. *H. concudense* is similar in most aspects to its geologically older relative cf. *H. depereti*. The epitympanic wing is a low-rounded structure protruding gently from the anterior portion of the promontorium. The caudal tympanic process, located posterior to the *fenestra cochlae*, is mediolaterally broad but not to the same extent as observed in *E. caballus* (O’Leary, 2010). The mastoid region is large, occupying about half the bone. As in both extinct and extant equids, the mastoid is shaped as a knob. Likewise, as in the other fossil equids described here, the mastoid region is incomplete, leaving only the fan-like proximal portion of the mastoid. The mastoid of cf. *H. concudense* is not as greatly expanded as that of cf. *H. depereti*. The thin bony lamina that covers the *hiatus Fallopii* is still preserved and separates the secondary facial foramen from the anterior hole of the *hiatus Fallopii* (Fig. 5B). The stapedial muscle fossa is extremely deep, large, and oval-shaped, although this may be related to allometry. The facial sulcus is deep.

In the ventrolateral or tympanic view, the promontorium is smooth, with no sign of a sulcus for the stapedial artery (Fig. 5B). The promontorium has a slight bulge to accommodate the cochlea within, like cf. *H. depereti* (Figs. 5C–5F). The *fenestra cochleae* is similar in shape and relative size to that of *E. caballus* (O’Leary, 2010), oval-shaped and smaller than the fenestra vestibuli. The *fenestra vestibuli* is more reniform than that of cf. *H. depereti* (Figs. 3B, 5B). The *crista interfenestralis* is broader than *E. caballus*, but not as broad as cf. *A. aurelianense* (Figs. 1A, 5B).

In dorsomedial view (Fig. 5E), the *tegmen tympani* is markedly smaller than *E. caballu*s, though slightly inflated laterally. The *tegmen tympani* has a flat surface and lacks the large anterior process seen on *E. caballus* (O’Leary, 2010). The basicapsular groove sits along the dorsomedial edge of the petrosal (Fig. 5D), and the cochlear aqueduct (Figs. 5E, 5F) sits in a slit situated more ventrally than ventromedially as in *E. caballus* (O’Leary, 2010). The vestibular aqueduct exits in a very similar position as in *E. caballus* on the dorsomedial face of the petrosal (Fig. 5E). In the anterior view (Fig. 5D), the superiormost portion of the petrosal does not project medially, at least not to the extent seen in *E. caballus* (O’Leary, 2010).

**Figure 6:**
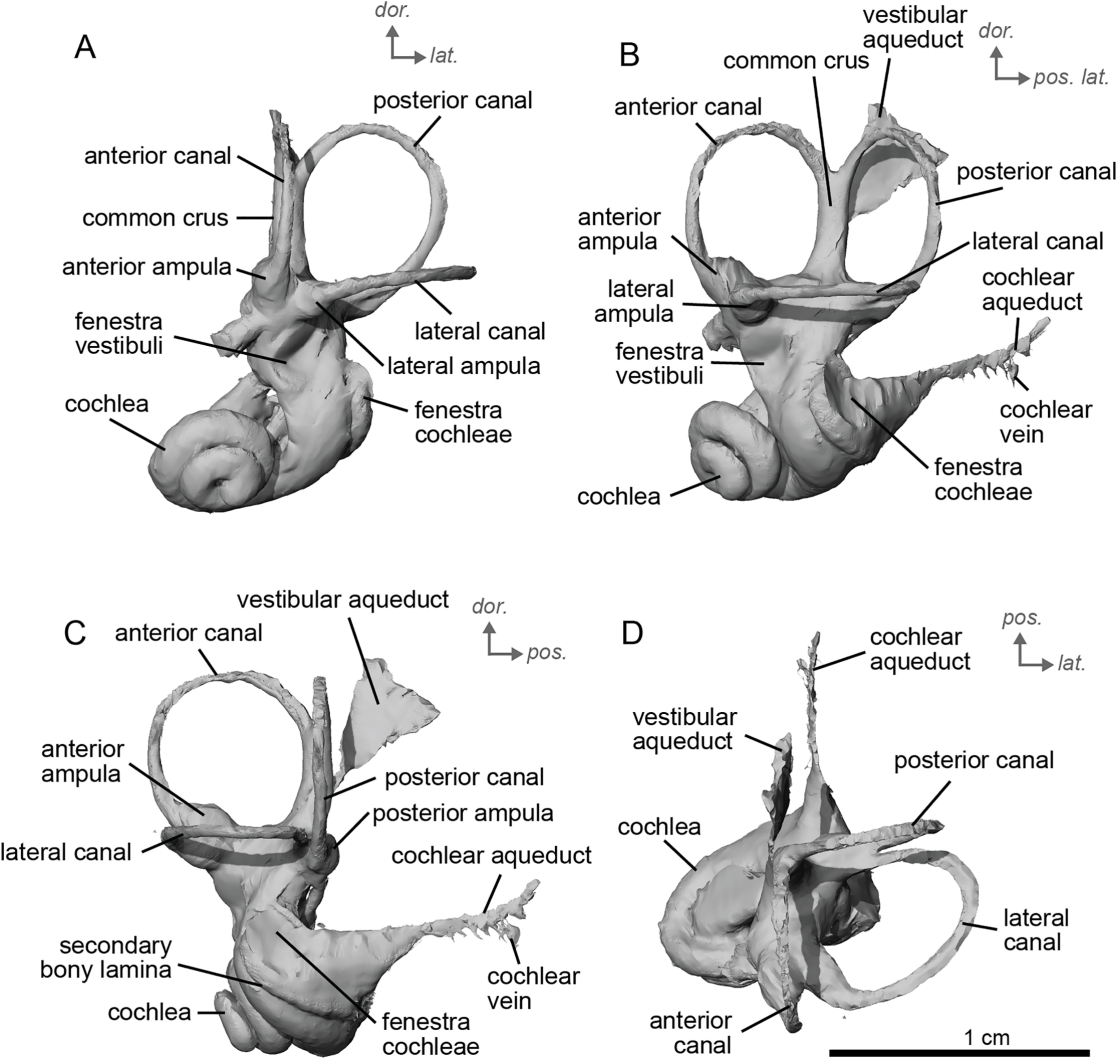
Endocast of the left bony labyrinth of cf. *H. concudense* (NMB.Ccd.3) from Concud 3. (A) Anterior view. (B) Posterior view. (C) Lateral view. (D) Dorsal view. *Dor*. = dorsal, *lat*. = lateral, *pos*. = posterior.

The bony labyrinth of cf. *H. concudense* is better preserved than that of cf. *H. depereti*. Like NMB.A.Mo.655, it shows little post-mortem deformation. A faint secondary bony lamina extends along the first 2/3rds of the basilar turn. The cochlea forms a loose spiral of 2.5 turns, like both cf. *H. depereti* and *E. caballus* (Ekdale, 2013). The cochlear aqueduct narrows as it nears the aperture of the petrosal, as observed in cf. *H. depereti*. The anterior semicircular canal has both the greatest height and radius of curvature. Like *E. caballus*, the posterior entry of the lateral semicircular canal is through the posterior ampulla, and the lateral semicircular canal attaches more dorsally to the vestibule than the posterior semicircular canal does. The lateral semicircular canal sits at a right angle relative to both the anterior and posterior semicircular canals. The angle between the posterior and anterior canal (Fig. 6D) is more obtuse than in cf. *H. depereti*. It also differs from cf. *H. depereti* by its more posteriorly elongated lateral canal in dorsal view (Fig. 6D). The anterior canal is more rounded than the posterior one. The lateral one is ovoid in shape rather than a semicircle. The long endolymphatic sac is triangular in shape and posteriorly projected. It starts below the common crus and runs mostly parallel to it. There is an inflection between the vestibule and the cochlea. The cochlea is weakly detached (short distance between the start of the first turn and the start of the second turn) from the vestibule. The vestibule of NMB.A.Mo.655 is well preserved enough that the sacculus and utriculus are visible. The sacculus and utriculus are well defined (Figs. 6C, 6D). The utriculus is bulbous and more pronounced than the sacculus. Like in *E. caballus*, the vestibular aqueduct is broad and forms a straight canal, but unlike *E. caballus*, the vestibular aqueduct is attached directly to the common crus, and not to the sacculus (Ekdale, 2013: fig. 32B).

**Figure 7:**
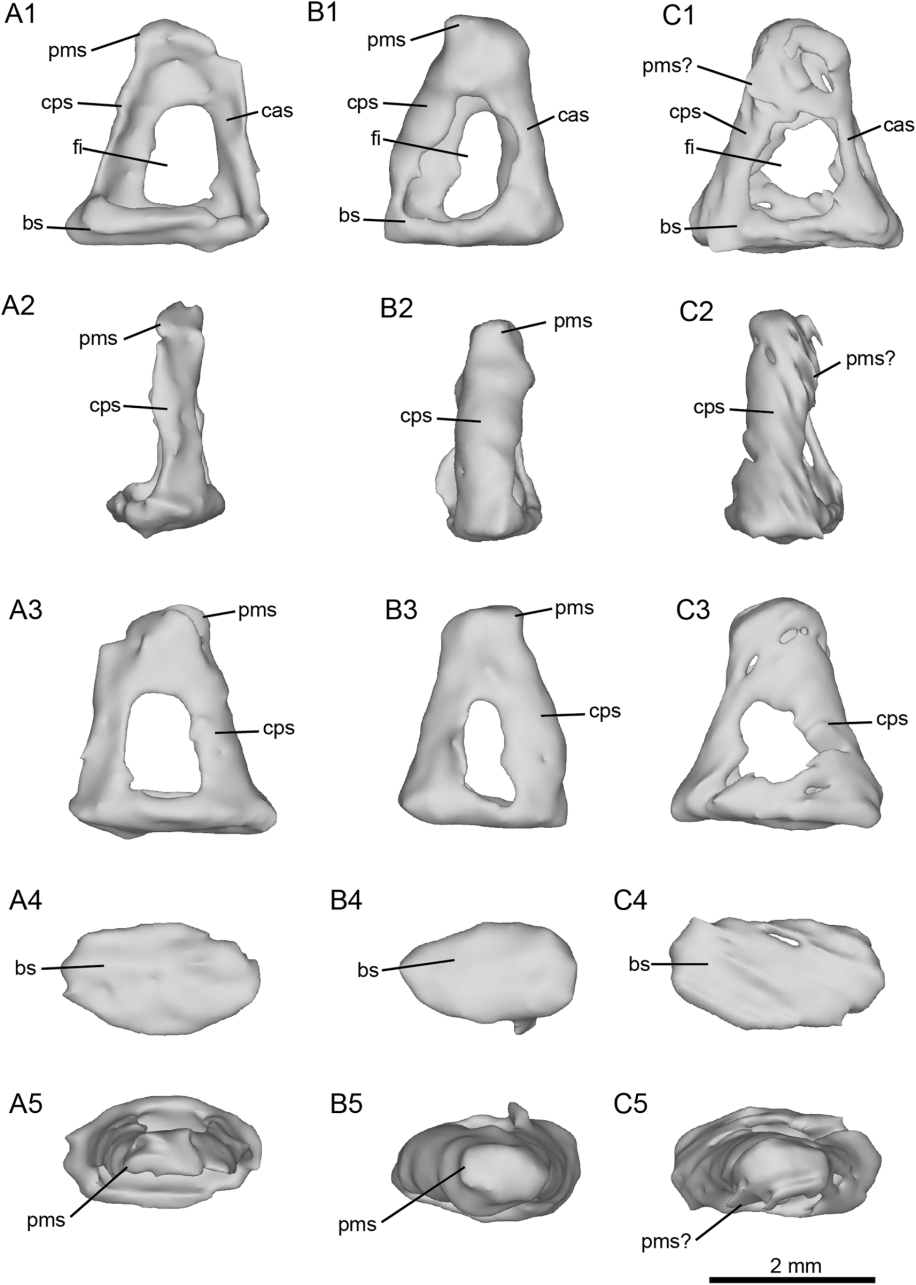
Left stapes preserved within the petrosals of fossil equids. (A) Cf. *Hipparion concudense* (NMB.Ccd.3). (B) Cf. *Equus stenonis* (NMB.V.A.2753). (C) Cf. *Equus senezensis* (NMB.Se.141). Abbreviations: bs, *basis stapedis*; cas, *crus anterius stapedis*; cps, *crus posterius stapedis*; fi, *foramen intercrurale*; pms, *processus muscularis stapedis*.

The stapes of cf. *H. concudense* (Fig. 7A) was preserved inside the vestibule of the bony labyrinth, which happens occasionally, as reported by Orliac & Billet (2016). It seems to be broken or poorly preserved, lacking most of its medial side (Fig. 7A). The general shape is quite similar to that of *E. caballus* illustrated by Doran (1878: pl. 61, fig. 3). In lateral view, the *foramen intercrurale* is large and rectangular. The *capitulum* cannot be distinguished from the rest of the body of the stapes or is not preserved. The *basis stapedis* is oval-shaped.

Equini Quinn, 1955

*Equus* Linnaeus, 1758

cf. *Equus stenonis* Cocchi, 1867

**Material**

An isolated left petrosal, NMB.V.A.2753

**Locality and Age**

Valdarno, Italy; Early Pleistocene, Villafranchian age (MNQ 18).

**Figure 8:**
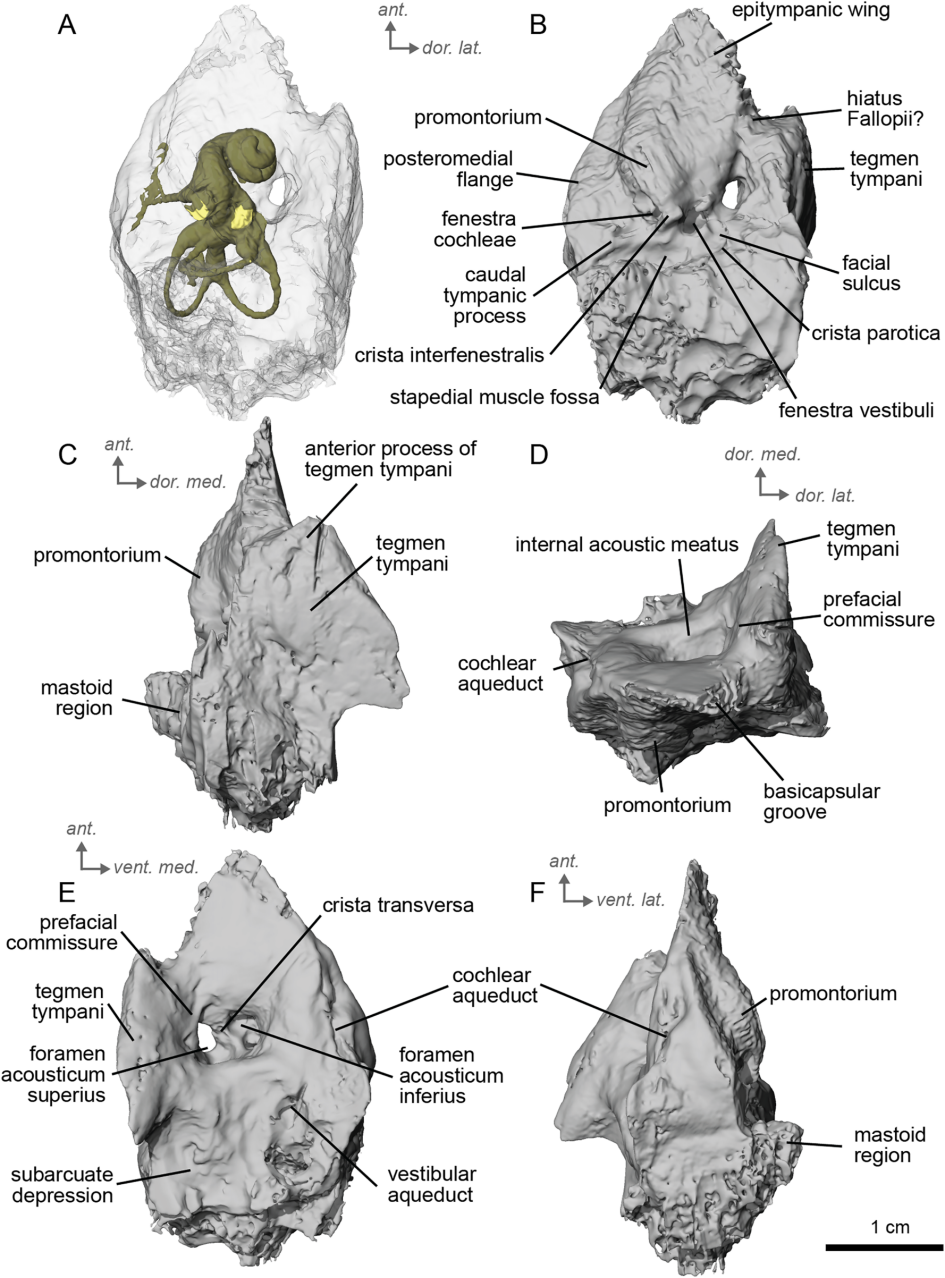
Left petrosal (NMB.V.A.2753) of cf. *Equus stenonis* from Valdarno. (A) Ventrolateral transparent view. (B) Ventrolateral opaque view. (C) dorsolateral view. (D) Anterior view. (E) Dorsomedial view. (F) Ventromedial view. *Ant* = anterior, *dor*. = dorsal, *lat*. = lateral, *med*. = medial, *vent*. = ventral.

**Description and comparison.** Cf. *Equus stenonis* from Valdarno is represented by an isolated petrosal (NMB.V.A.2753) measuring 3.55 cm in length. The petrosal is well preserved, with the only major damage being to the mastoid region. The Valdarno *Equus* preserved a stapes within the bony labyrinth. In overall character, the petrosal is strikingly more similar to *E. caballus* (O’Leary, 2010) and cf. *E. senezensis* than to *Hipparion* and especially cf. *Anchitherium*. Cf. *E. stenonis* and cf. *E. senezensis* are similar to *E. caballus* in possessing a prominent anterior process of the *tegmen tympani*, while *Hipparion* and cf. *Anchitherium* have low *tegmina tympani* lacking such a pronounced anterior process. This anterior process ends in a point, as in *E. caballus* (O’Leary, 2010). The anterior process of the *tegmen tympani* of cf. *E. stenonis* does not extend anterior to the promontorium. The caudal tympanic process is wide and smooth (Fig. 8B), with an expansion similar to extant *E. caballus* rather than *Hipparion* or cf. *Anchitherium*. The promontorium is barely distinguished but seems elongated and bordered by a long posteromedial flange. The *fenestrae vestibuli* and *cochleae* are of similar size. The mastoid region is large, with a broad proximal area like the other equids in this study. The subarcuate depression is wide and shallow as in *E. caballus* (O’Leary, 2010). The vestibular aqueduct opens in a slit lateral to the subarcuate depression and posteromedial to the cochlear aqueduct. The mastoid meets and joins the caudal tympanic process such that the mastoid juts out at an angle when viewed in ventrolateral view (Fig. 8F). Two grooves along the ventromedial edge of the bone demarcate the basicapsular groove (Figs. 8D, 8E), with the cochlear aqueduct laying at the posterior edge of this groove. The ventrolateral side (Fig. 8B) is not very well preserved, and the segmentation of this area was difficult due to the preservation of the bulla, which we excluded in the figures. The *hiatus Fallopii* is not well preserved but may lie between the epitympanic wing and the *tegmen tympani* (Fig. 8B). The facial sulcus is barely visible and quite shallow. The petrosal is narrow in ventromedial view, expanding into a fan-shaped mastoid process (Fig. 8F). The promontorium is barely distinguished but seems elongated and bordered by a long posteromedial flange. The *fenestrae vestibuli* and *cochleae* are similar-sized.

**Figure 9:**
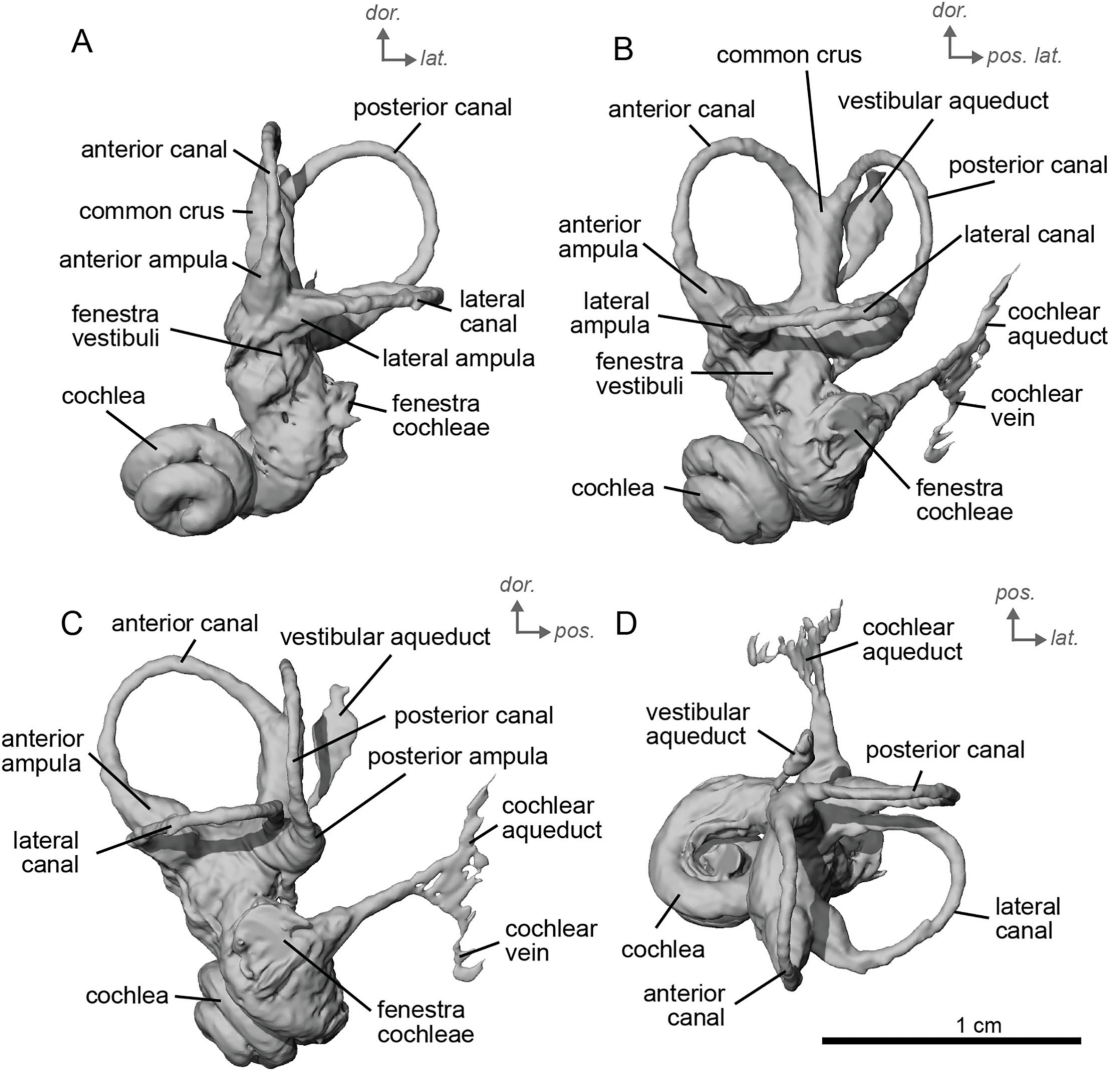
Endocast of the left bony labyrinth of cf. *Equus stenonis* (NMB.V.A.2753) from Valdarno. (A) Anterior view. (B) Posterior view. (C) Lateral view. (D) Dorsal view. *Dor*. = dorsal, *lat*. = lateral, *pos*. = posterior.

The bony labyrinth of cf. *E. stenonis* is nearly identical to *E. caballus* (Ekdale, 2013). The cochlea is loosely coiled, forming 2.5 turns. The cochlea takes on an elliptical shape, longer anteroposteriorly than mediolaterally. A faint bony lamina extends along the first 2/3rds of the basilar turn of the cochlea (Fig. 9). Like *E. caballus* the posterior entry of the lateral semicircular canal is through the posterior ampulla (Fig. 9C), the largest semicircular arc radius of curvature is in the anterior semicircular canal, and the lateral semicircular canal attaches more dorsally to the vestibule than the posterior semicircular canal does (Fig. 9C). The posterior ampulla is larger than in *Hipparion* (Figs. 9B, 9C). However, the anterior canal seems to be more elliptic than the posterior one due to a straight projection of the anterior canal when connecting with the common crus (Fig. 9B). The lateral canal is slightly ovoid in shape (Fig. 9D) and larger than in cf. *H. concudense*. The small endolymphatic sac is triangular in shape and posteriorly projected, not in line with the common crus (Fig. 9B). It starts close to the base of the common crus due to a very short and posteriorly projected vestibular aqueduct (Fig. 9C). There is an inflection between the vestibule and the cochlea. The cochlea is weakly detached (short distance between the start of the first turn and the start of the second turn) from the vestibule, with a relatively short distance between the vestibule and cochlear coils (Fig. 9A). The cochlear vein is preserved (Figs. 9B, 9C), ventral to the cochlear aqueduct, as in *E. caballus* (Ekdale, 2013).

The stapes of cf. *E. stenonis* (Fig. 7B) is very similar to that of cf. *H. concudense*, but it is more complete. The *foramen intercrurale* is larger on the lateral side than on the medial side, as observed in some artiodactyls (Orliac & Billet, 2016). The *basis stapedis* is oval-shaped (Fig. 7B). In medial and lateral views (Fig. 7B), the stapes is roughly triangular. The *capitulum stapedis* cannot be differentiated from the rest of the body of the stapes. In medial view, the *foramen intercrurale* is much smaller than on the lateral side.

cf. *Equus senezensis*, Azzaroli, 1965

**Material**

Three petrosals, including the left and right petrosals and one stapes of a single individual (NMB.Se.141) and an isolated right petrosal (NMB.Se.554).

**Locality and Age**

Senèze, France; Early Pleistocene, Villafranchian (MNQ 18).

**Figure 10:**
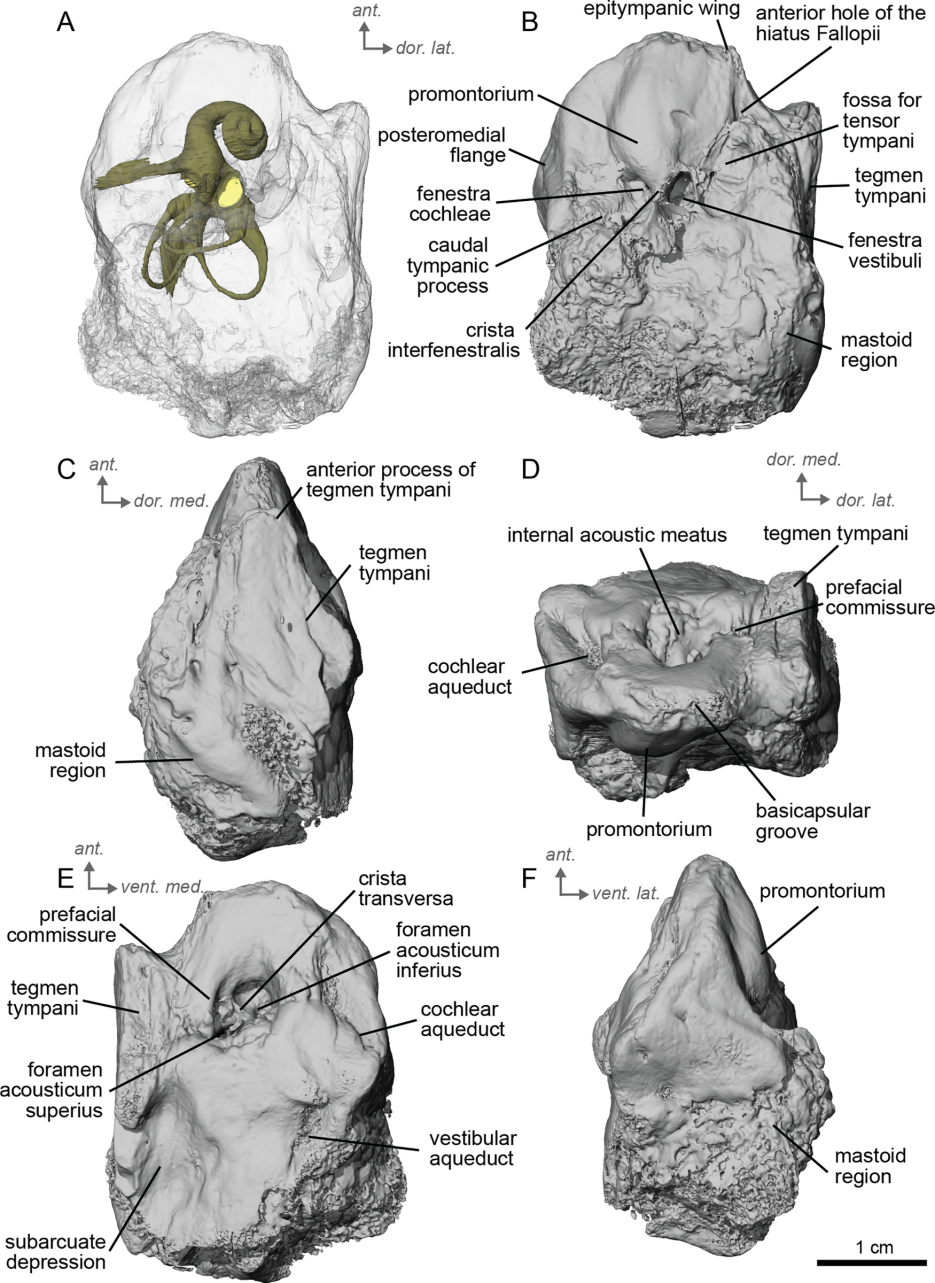
Left petrosal (NMB.Se.141) of cf. *Equus senezensis* from Senèze. (A) Ventrolateral transparent view. (B) Ventrolateral opaque view. (C) Dorsolateral view. (D) Anterior view. (E) Dorsomedial view. (F) Ventromedial view. *Ant* = anterior, *dor*. = dorsal, *lat*. = lateral, *med*. = medial, *vent*. = ventral.

**Figure 11:**
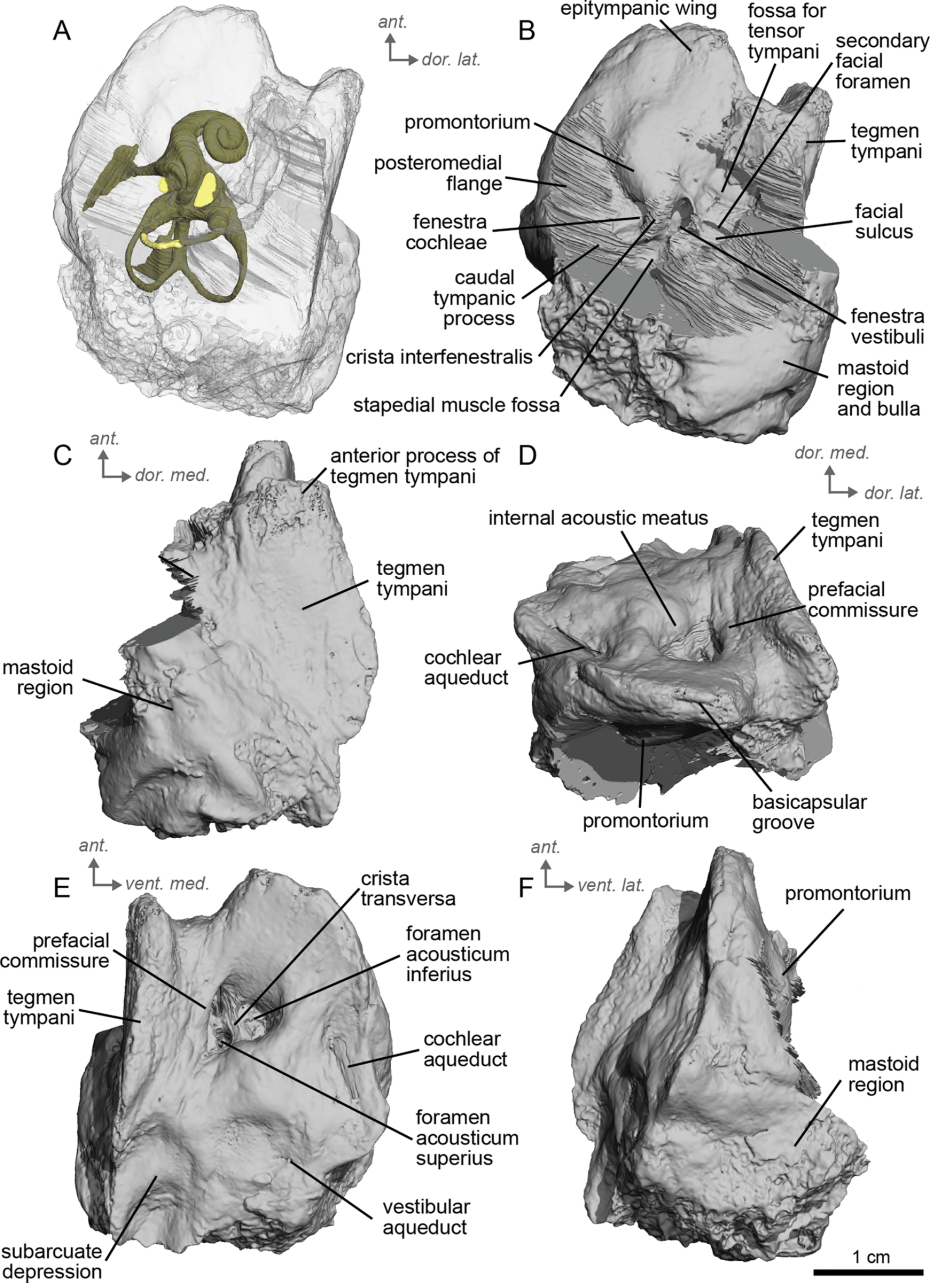
Right petrosal (NMB.Se.554) of cf. *Equus senezensis* from Senèze (mirrored). (A) Ventrolateral transparent view. (B) Ventrolateral opaque view. (C) Dorsolateral view. (D) Anterior view. (E) Dorsomedial view. (F) Ventromedial view. *Ant* = anterior, *dor*. = dorsal, *lat*. = lateral, *med*. = medial, *vent*. = ventral.

**Description & comparison.** Cf. *Equus senezensis* was represented by three petrosals, two of which represent the left and right petrosals of a single individual, NMB.Se.141, and one from NMB.Se.554. The petrosals vary in size from 3.60 to 3.94 cm anteroposteriorly. They differ from that of *E. caballus* (O’Leary, 2010) in several ways. Between the *fenestra cochleae* and the *fenestra vestibuli* is a *crista interfenestralis* that is pronounced, but less distinct than in *E. caballus* (O’Leary, 2010). The fossa for the *tensor tympani* muscle is a large, oval, and deep depression (Fig. 10D). It significantly excavates the surrounding *tegmen tympani* (Fig. 10B). The epitympanic wing is present and protrudes from the *promontorium* but is very small (Fig. 10B). There is a distinct anterior hole for the *hiatus Fallopii* and it is relatively large (Fig. 10B). A posteromedial flange extends from the *promontorium* such that the *promontorium* is surrounded by a complete, flat flange of bone similar to that of *E. caballus* (O’Leary, 2010), though it appears to extend even further posteriorly. On the second specimen (NMB.Se.554), the fossa for the *tensor tympani* muscle is shallower, and oval (Fig. 11B). The *tegmen tympani* is flat and moderately inflated, contributing to about one-fifth the total width of the ventrolateral view though slightly more inflated than that of *E. caballus* (O’Leary, 2010). The anterior process of the *tegmen tympani* is larger than that of *E. caballus* and extends anterior to the promontorium before terminating in a less pronounced point (O’Leary, 2010). The mastoid region is large and wedge-shaped, irregular, and knobby, though it appears rounder than that of *E. caballus* (O’Leary, 2010). The facial sulcus and stapedial muscle fossa cannot be observed.

The ventromedial edge of the bone has a basicapsular groove, and at its posterior edge houses a small cochlear aqueduct (Fig. 11E). The slit for the cochlear aqueduct is smaller in NMB.Se.554 (Fig. 11E) than in NMB.Se.141 (Fig. 10E). The vestibular aqueduct opens in a slit lateral to the subarcuate depression and posteromedial to the cochlear aqueduct (Figs. 10E, 11E). In the ventromedial view, the petrosal is narrow and widens into a fan-shaped mastoid region with bumps and projections. The ventromedial surface is relatively flat (Fig. 11C). There are vascular grooves on the dorsolateral side of the *tegmen tympani* (Fig. 10C). The cochlear aqueduct is a small hole at the ventromedial margin but is found within a large slit (Figs. 10D, 10E).

We can observe patterns of intra-individual variation due to asymmetry in the petrosal of cf. *E. senezensis*. The two petrosals of NMB.Se.141 differ indeed in some respects, such as the subarcuate depression, which is smaller in the right petrosal than in the left one (see additional specimen available on Morphosource project: https://www.morphosource.org/projects/000720375?locale=en). Such variation is consistent with Danilo et al.’s 2015 study of modern *Equus*, where the size, morphology, elongation, and depth of the subarcuate depression can be highly variable among individuals.

**Figure 12:**
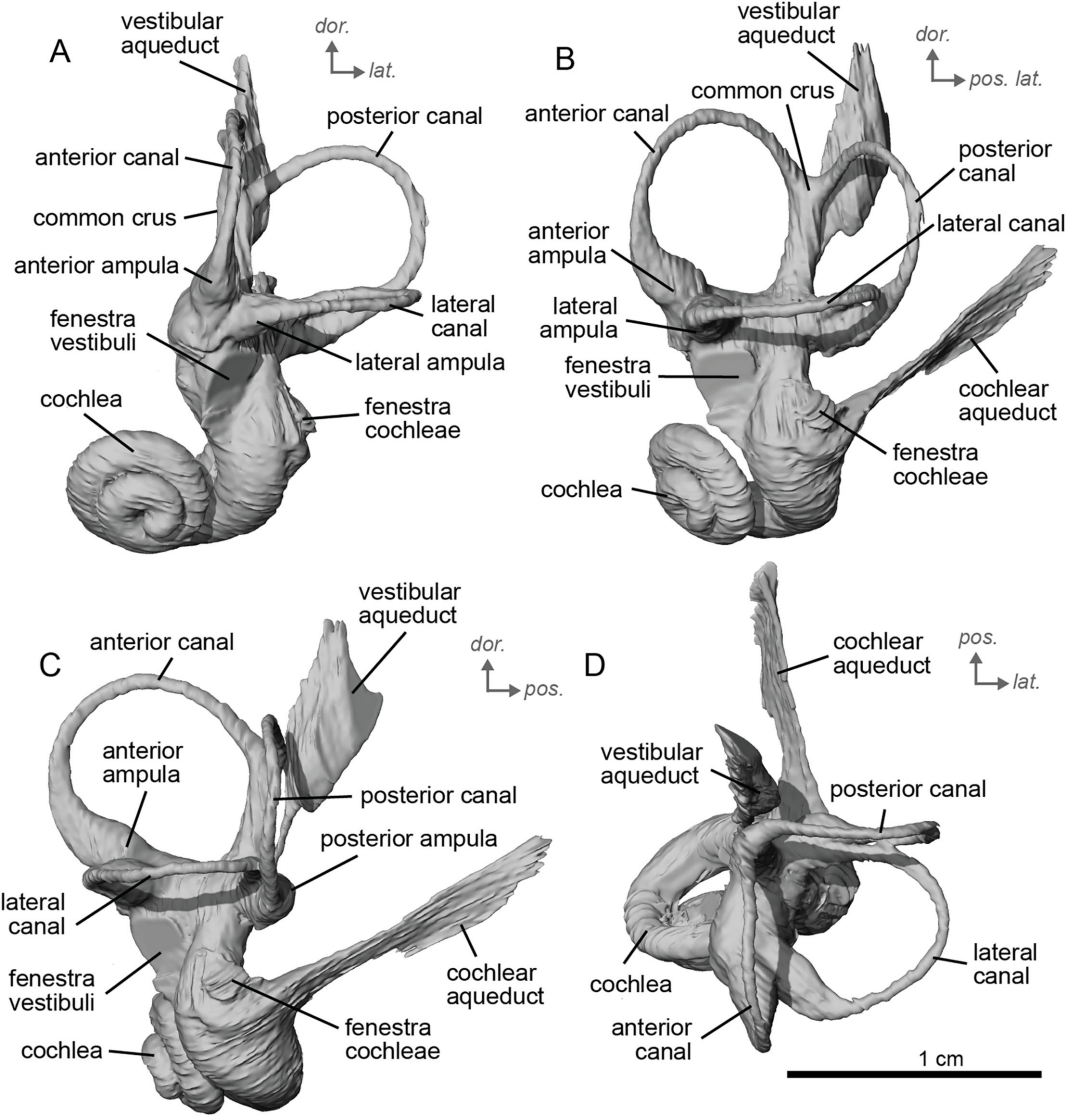
Endocast of the left bony labyrinth of cf. *Equus senezensis* (NMB.Se.141) from Senèze. (A) Anterior view. (B) Posterior view. (C) Lateral view. (D) Dorsal view. *Dor*. = dorsal, *lat*. = lateral, *pos*. = posterior.

**Figure 13:**
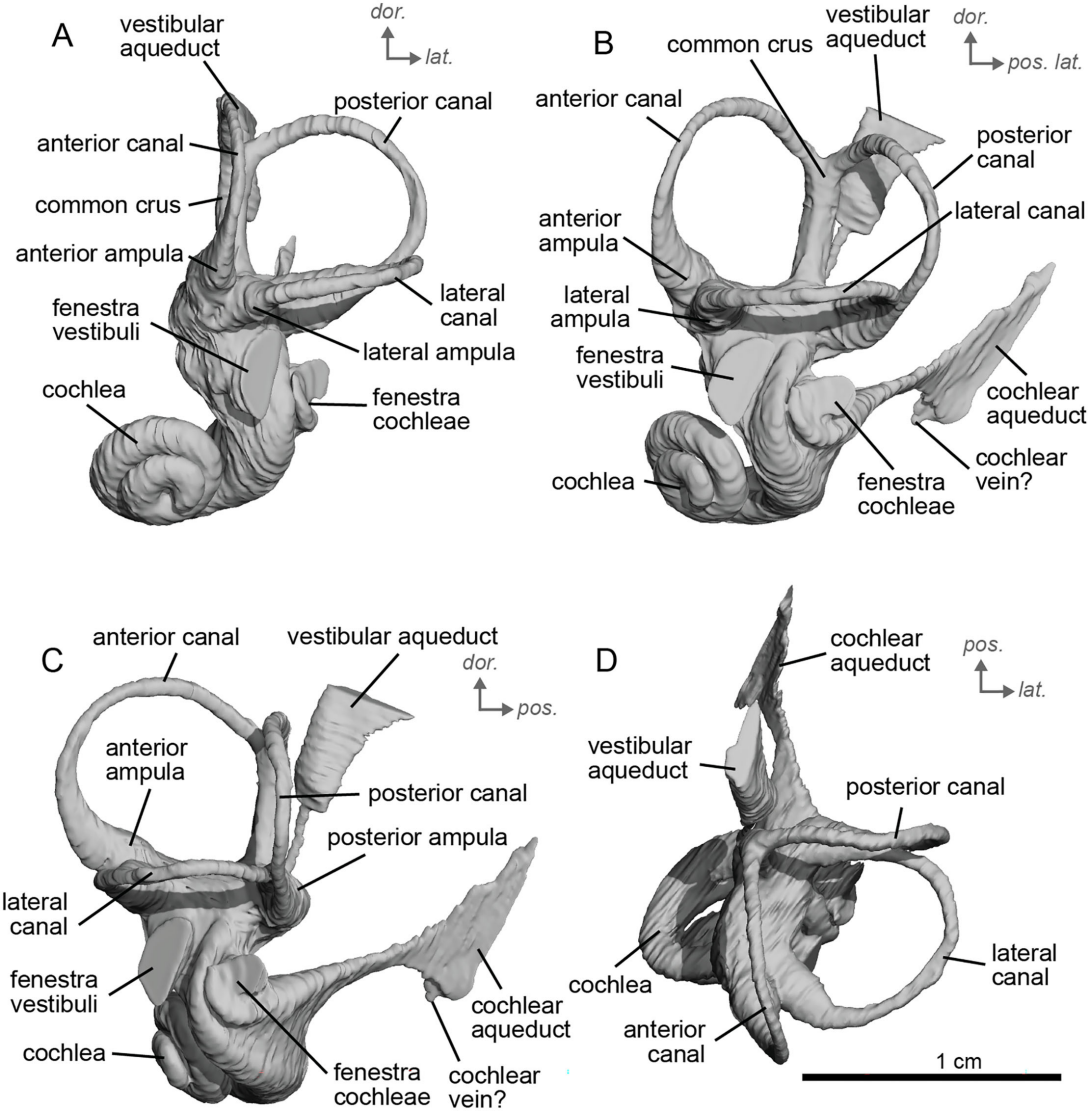
Endocast of the right bony labyrinth of cf. *Equus senezensis* (NMB.Se.554) from Senèze (mirrored). (A) Anterior view. (B) Posterior view. (C) Lateral view. (D) Dorsal view. *Dor*. = dorsal, *lat*. = lateral, *pos*. = posterior.

The basal portion of the cochlear coil of cf. *E. senezensis* (NMB.Se.141 and NMB.Se.554; Figs. 12, 13) has a straight proximal section which causes the cochlear spiral to appear more separate from the vestibule. The latter part of the coil begins as a loose spiral that becomes tighter towards the apex. The coil remains tightly wound consistently after about the first quarter of the first basal turn. The cochlea completes about 2.5 turns (Figs. 12A, 13A) and is more tightly coiled than *E. caballus* (Ekdale, 2013). The cochlea of cf. *E. senezensis* has an aspect ratio ranging from low (0.44) to high (0.58; Table 2), in contrast to the low aspect ratio of *E. caballus* (0.41; Ekdale, 2013, tab. 2). There is no secondary bony lamina on the cochlea of either the left or right bony labyrinth of NMB.Se.141 (Figs. 12A, 13A), but NMB.Se.554 does preserve a faint secondary bony lamina like cf. *E. stenonis*, and *E. caballus* (Ekdale, 2013).

NMB.Se.554 has the spherical and elliptical recesses separated by a narrowing of the vestibule. This condition is observed in both *E. caballus* (Ekdale, 2013) and cf. *E. senezensis*, but is more pronounced in this individual than in NMB.Se.141. The entry of the lateral semicircular canal into the posterior one is through the posterior ampulla (Figs. 12B, 13B). The bony ring is quite pronounced and nearly parallel to the plane of the lateral semicircular canal. The posterior canal seems to be more elliptic than the anterior one. The lateral one is relatively rounded (Figs. 12D, 13D). The large endolymphatic sac is rectangular in shape and posteriorly projected, not in line with the common crus (Figs. 12B, 13B). It starts almost at mid-height of the common crus due to a slightly posteriorly projected vestibular aqueduct. There is an inflection between the vestibule and the cochlea. The cochlea is weakly detached (short distance between the start of the first turn and the start of the second turn) from the vestibule.

The stapes of cf. *E. senezensis* was preserved inside the vestibule of the left petrosal NMB.Se.141 (Fig. 7C). It is overall quite similar to *E. caballus* (Doran, 1878), or the other two stapes described here, but is not well preserved. The *processus muscularis stapedis* is difficult to identify, but seems to be visible on the posterolateral face of the stapes (Fig. 7C). As in the other species, the *foramen intercrurale* is smaller on the medial side than on the lateral side. The *capitulum* cannot be identified.

### Preliminary phylogenetic analysis

**Figure 14:**
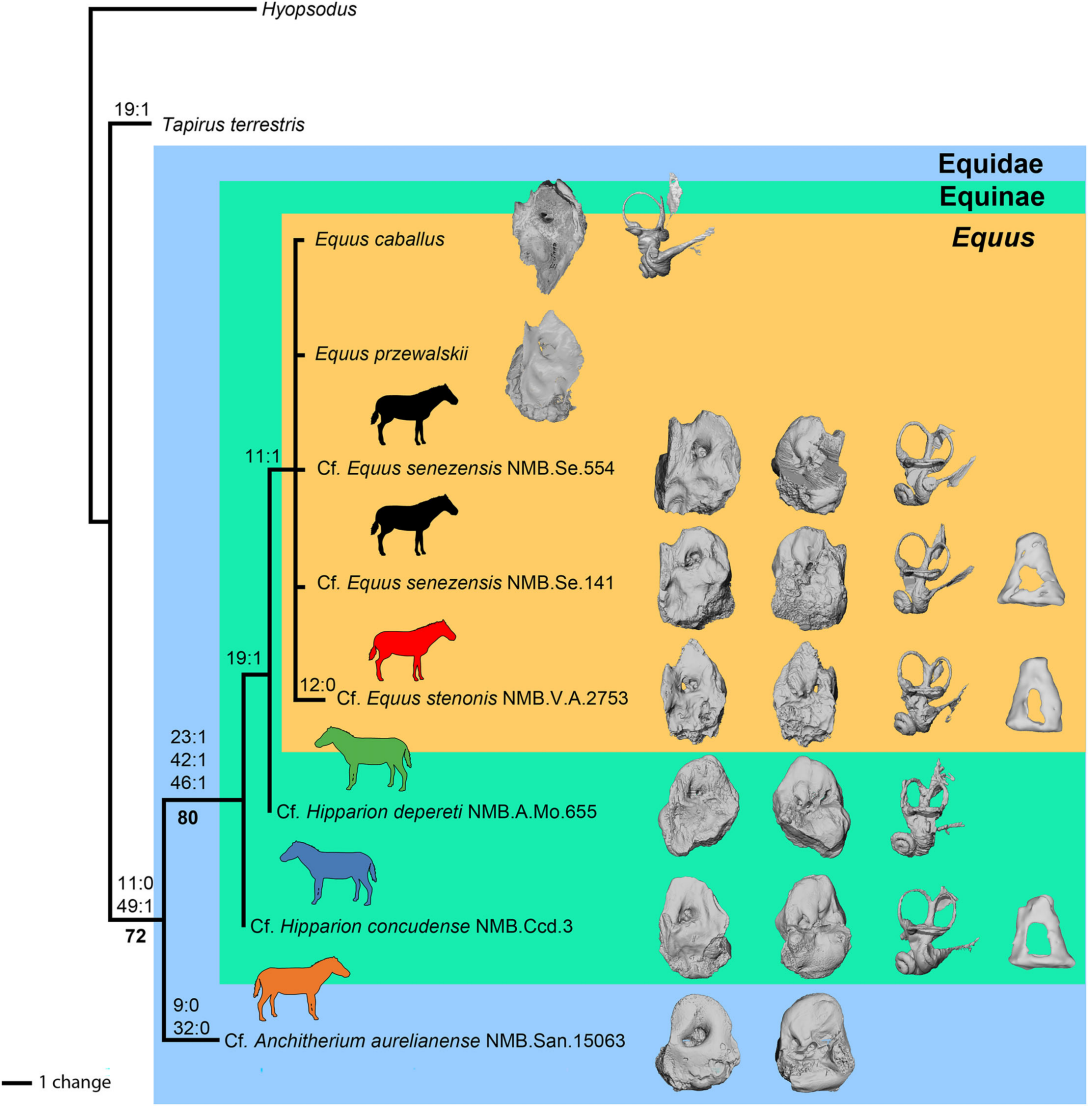
Phylogeny of the studied Equidae based on ear region characters. Strict consensus tree of 11 trees of 24 steps (CI = 0.88, HI = 0.13, RI = 0.80) with *Hyopsodus* used as outgroup, obtained by an exhaustive search with a parsimony algorithm in PAUP∗4. Non-ambiguous apomorphies are indicated as “character number:state” at nodes. Bootstrap support values above 50 are reported in bold font below nodes. Image Source Credit: Zimices, https://www.phylopic.org/images/0df28002-4d73-4679-95c0-7f271e4c89c1/equus-stenonis, CC BY-SA 4.0.

Using our combined dataset (see Material and Methods), we obtained 11 most-parsimonious trees of 24 steps, with a consistency index (CI) of 0.88, a retention index (RI) of 0.80, and a homoplasy index (HI) of 0.13. These 11 trees are included in the nexus file provided in File S1. The strict consensus tree is shown in Fig. 14. Of the 50 characters in the analysis, 29 are constant and only nine are parsimony informative. The clade containing the Equidae in this study is supported by six (ambiguous and non-ambiguous) synapomorphies in ACCTRAN optimization:

- the absence of anterior process of the *tegmen tympani*,
- the apex of the anterior process of the *tegmen tympani* is large (when it is present); the position of this synapomorphy is ambiguous
- the apex of the anterior process of the *tegmen tympani* pointed (when it is present); the position of this synapomorphy is ambiguous
- the ventrolateral tuberosity of petrosal is present; the position of this synapomorphy is ambiguous
- the lateral semicircular canal attaches more dorsally to the vestibule than the posterior canal; the position of this synapomorphy is ambiguous
- and the cochlear aqueduct is on the ventral face of the petrosal.

The clade Equinae is supported by three synapomorphies:

- the pars cochlearis protrudes ventromedially,
- the caudal tympanic process is long,
- and the subarcuate depression is wide.

Cf. *Anchitherium* has two autapomorphies, whereas cf. *E. stenonis* has one. *Equus* is monophyletic and supported by one unambiguous synapomorphy in ACCTRAN optimization: the presence of an anterior process of the *tegmen tympani* (character 11). All perissodactyls, including *Tapirus*, differ from *Hyopsodus* by eight characters (characters 3, 20, 24, 25, 27, 29, 43 and 50). In our analysis, *Hipparion* is paraphyletic, and cf. *H. concudense* differs from cf. *H. depereti* by the absence of extension of the fossa for the *tensor tympani* muscle.

## Discussion

In both extant tapirs and horses, characters of the petrosal have been observed to be variable among individuals. Among equids, Danilo et al. (2015) described the variability of 14 petrosals of *E. przewalskii* belonging to individuals of varying ages, sexes, and sizes. These differences have also been observed in artiodactyls, such as an ontogenetic series of *Bos* (Costeur et al., 2017). They showed that the depth of the petrosal groove, the shape of the internal acoustic meatus, as well as the shape, size, and elongation of the subarcuate depression were individually variable depending on the age of the individual. Mateus (2018) showed that in *Tapirus terrestris*, the depth and size of the subarcuate depression, as well as the position of the caudal tympanic process and the *hiatus Fallopii*, are individually variable as in *E. przewalskii*. The subarcuate depression accounted for three characters in Spaulding, O’Leary & Gatesy (2009) and one in Mateus (2018). All the perissodactyls in our analysis shared a shallow subarcuate depression. *Tapirus terrestris* and cf. *A. aurelianense* were both scored as small for the size of the subarcuate depression while all the other perissodactyl taxa were scored as large.

The petrosal bone, contrary to the bony labyrinth, is a structure that ossifies in parallel to the surrounding skull bones (Mennecart & Costeur, 2016, Costeur et al., 2017). Because of this, this bone may greatly suffer from allometry (ontogenic and evolutionary), complexifying the interpretation of the evolutionary polarity of the characters. The bony labyrinth is a cavity that fully ossifies during fetal stages in placental mammals (*e.g*., Mennecart & Costeur, 2016; Costeur et al., 2017). Shape and size remain similar during the life of the animal, allowing it to provide morphological characters that are not sensitive to variation over ontogeny (Evin et al., 2022; Mennecart et al., 2022). This organ may represent a structure with a neutral evolution in artiodactyls (Mennecart et al., 2022), where gradual changes can be observed.

The ear region of extinct perissodactyls remains poorly understood, relative to artiodactyls. The petrosal of extinct perissodactyls was only described for three equoids (Kitts, 1956; Cifelli, 1982; O’Leary, 2010), four tapiromorphs (Radinsky, 1965; Colbert, 2006; Ping & Yuan-Qing, 2010; O’Leary, 2010), one brontothere (Mader, 2009), and one ancylopod (Bai, Wang & Meng, 2010) so far. The petrosal of rhinocerotoids is especially poorly understood. O’Leary (2010) briefly described the incomplete petrosal of *Dicerorhinus sumatrensis* in their morphological characters matrix, while (Robert et al., 2021) investigated the petrosal and bony labyrinth of *Ceratotherium simum simum*, and Manning, Dockery & Schiebout (1985) reported an isolated petrosal of *Metamynodon*.

Moreover, recent molecular investigations have suggested that the extinct South American Native Ungulates (SANUs) were related to perissodactyls (Welker et al., 2015) but comparisons between the petrosals of SANUs and other placentals have been limited thus far, and have not included perissodactyls (Billet & Muizon, 2013). The contribution of the ear region to the phylogeny of perissodactyls may, therefore, also be key to illuminating their potential relationships to SANUs and other extinct hoofed mammal groups like phenacodontids and cambaytheres.

## Conclusions

The result of our preliminary phylogenetic analysis suggests that the ear region is informative for perissodactyl phylogeny and invites future research. This limited analysis suggests that the petrosal morphology may be informative in family and genus-level cladistics, but it currently lacks precision for detecting generic-level distinctions, considering its inability to recover the monophyly of the genus *Hipparion*. Yet, it is also possible that the two species cf. *H. depereti* and cf. *H. concudense* do not belong to the same genus, since *Hipparion* may actually be paraphyletic according to another phylogenetic analysis (Sun et al., 2025). Further investigation is necessary to better understand the phylogenetic utility of the selected character in regards to their allometric constrains and variation. Moreover, the bony labyrinth has been proven to be a structure that evolves mostly neutrally in ruminants. Including more bony labyrinth characters in future phylogenetic analyses may be useful for developing a better understanding of the perissodactyls’ evolutionary history and for obtaining more refined results. Obviously, a much larger sampling would be needed to fully investigate the phylogeny of Equidae or other perissodactyls, but we believe that the petrosal and inner ear’s morphology could be a valuable addition in future larger-scale phylogenetic analysis. Although only nine characters were parsimony informative, we think that with a more diverse taxonomic sample, more characters would become phylogenetically informative.

## Supplemental Information

10.7717/peerj.20484/supp-1 Supplemental Information 1 Morphological data matrix including the 11 most parsimonious trees.

10.7717/peerj.20484/supp-2 Supplemental Information 2 Characters list.

## Additional Information and Declarations

### Data Availability

The following information was supplied regarding data availability:

The data is available in the Supplemental Files.

The tomograms and 3D models of petrosals, bony labyrinths, and stapes are available at Morphosource:

- https://www.morphosource.org/projects/000720375?locale=en.

- https://doi.org/10.17602/M2/M722307.

- https://doi.org/10.17602/M2/M722303.

- https://doi.org/10.17602/M2/M722294.

- https://doi.org/10.17602/M2/M722283.

- https://doi.org/10.17602/M2/M722253.

- https://doi.org/10.17602/M2/M722224.

- https://doi.org/10.17602/M2/M722167.

- https://doi.org/10.17602/M2/M722164.

- https://doi.org/10.17602/M2/M722157.

- https://doi.org/10.17602/M2/M722145.

- https://doi.org/10.17602/M2/M721988.

- https://doi.org/10.17602/M2/M721974.

- https://doi.org/10.17602/M2/M721968.

- https://doi.org/10.17602/M2/M721965.

- https://doi.org/10.17602/M2/M721950.

- https://doi.org/10.17602/M2/M721947.

- https://doi.org/10.17602/M2/M721939.

- https://doi.org/10.17602/M2/M721927.

- https://doi.org/10.17602/M2/M721919.

- https://doi.org/10.17602/M2/M721916.

- https://doi.org/10.17602/M2/M720386.

- https://doi.org/10.17602/M2/M720383.
