# Supplementary material for: The petrosal and bony labyrinth of extinct horses (Perissodactyla, Equidae) and their implications for perissodactyl evolution
Source: PeerJ. 2026 Jan 5;14:e20484. doi: 10.7717/peerj.20484 (PMC12782039; doi:10.7717/peerj.20484)
Supplement: Supplemental Information 2 [file peerj-14-20484-s002.docx]

Character list

1. Petrosal: texture of Area around internal acoustic meatus; O’Leary & Gatesy 2008 c1
   - States
     1. rough
     2. smooth
2. Petrosal: Subarcuate fossa; O’Leary & Gatesy 2008 c2
   - States
     1. present
     2. absent
3. Petrosal: Subarcuate fossa depth; O’Leary & Gatesy 2008 c3
   - States
     1. deep
     2. shallow
4. Petrosal: Petromastoid canal in subarcuate fossa; O’Leary & Gatesy 2008 c4
   - States
     1. absent
     2. present
5. Petrosal: Hiatus Fallopii distinct anterior hole; O’Leary & Gatesy 2008 c5
   - States
     1. present
     2. absent
6. Petrosal: Hiatus Fallopii, size if foramen is positioned anteriorly; O’Leary & Gatesy 2008 c6
   - States
     1. small
     2. large
7. Petrosal: Prefacial commissure fossa of the petrosal; O’Leary & Gatesy 2008 c7
   - States
     1. absent (convex, flat or a bump)
     2. present (concave)
8. Petrosal: Shape of tegmen tympani; O’Leary & Gatesy 2008 c8
   - States
     1. flat
     2. pronounced convexity
9. Petrosal: Inflation of the tegmen tympani; O’Leary & Gatesy 2008 c9
   - States
     1. absent
     2. present
10. Petrosal: Degree of inflation of the tegmen tympani; O’Leary & Gatesy 2008 c10
    - States
      1. hyperinflation (transverse width > or = width of promontorium)
      2. moderate (1/2-1/4 width of promontorium)
11. Petrosal: Anterior process of the tegmen tympani; O’Leary & Gatesy 2008 c11
    - States
      1. absent
      2. present
12. Petrosal: Size of anterior process of the tegmen tympani; O’Leary & Gatesy 2008 c13
    - States
      1. small
      2. large, projects anterior to promontorium
13. Petrosal: Apex of anterior process of tegmen tympani – shape; O’Leary & Gatesy 2008 c12
    - States
      1. pointed
      2. blunt
14. Petrosal: Vascular groove on the lateral surface of the tegmen tympani; O’Leary & Gatesy 2008 c14
    - States
      1. present
      2. absent
15. Petrosal: Distinct fossa for the head of the malleus on petrosal; O’Leary & Gatesy 2008 c15
    - States
      1. absent
      2. present
16. Petrosal: Ventrolateral tuberosity of the petrosal; O’Leary & Gatesy 2008 c16
    - States
      1. absent
      2. present
17. Petrosal: Shape of ventrolateral tuberosity of petrosal; O’Leary & Gatesy 2008 c17
    - States
      1. bump/knob
      2. spike
18. Petrosal: Fossa for tensor tympani muscle round or oval depression; O’Leary & Gatesy 2008 c18
    - States
      1. present
      2. absent
19. Petrosal: Fossa for tensor tympani (if round or oval) extends into an excavated portion of the tegmen tympani; O’Leary & Gatesy 2008 c19
    - States
      1. absent
      2. present
20. Petrosal: Transpromontorial sulcus; O’Leary & Gatesy 2008 c20
    - States
      1. present
      2. absent
21. Petrosal: Double transpromontorial sulci; O’Leary & Gatesy 2008 c21
    - States
      1. absent
      2. present
22. Petrosal: Sulcus for stapedial artery on promontorium; O’Leary & Gatesy 2008 c22
    - States
      1. present
      2. absent
23. Petrosal: Pars cochlearis protrudes ventromedially; O’Leary & Gatesy 2008 c23
    - States
      1. absent
      2. present
24. Petrosal: Mastoid region size relative to promontorium; O’Leary & Gatesy 2008 c25
    - States
      1. small (~ 50%)
      2. large (~100% or more)
25. Petrosal: Shape of bone of mastoid region of the petrosal; O’Leary & Gatesy 2008 c26
    - States
      1. square, robust knob
      2. wedge
26. Petrosal: Epitympanic wing; Spaulding et al. 2009 c26
    - States
      1. absent
      2. present
27. Petrosal: Posteromedial flange of promontorium; O’Leary & Gatesy 2008
    - States
      1. absent
      2. present
28. Petrosal: Shape of promontorium; O’Leary & Gatesy 2008
    - States
      1. hemi-ellipsoid
      2. hemi-spherical
      3. 2=almond-shaped
29. Petrosal: Caudal tympanic process shape; O’Leary & Gatesy 2008
    - States
      1. mediolaterally narrow, bar of bone medial to stapedial muscle fossa
      2. mediolaterally broad, sometimes with irregular projections or knobs
30. Petrosal: Mastoid plate; O’Leary & Gatesy 2008 c28
    - States
      1. absent
      2. present
31. Petrosal: Basicapsular groove on pars cochlearis of the petrosal; Spaulding et al. 2009 c31
    - States
      1. present
      2. absent
32. Petrosal: Position of basicapsular groove; Spaulding et al. 2009 c32
    - States
      1. ventral
      2. dorsal
33. Petrosal: Secondary facial foramen position (relative to fenestra cochleae and fenestra vestibuli); O’Leary & Gatesy 2008 c30
    - States
      1. anterior
      2. posterior
34. Stapes: presence of stapedial foramen; O’Leary & Gatesy 2008 c60
    - States
      1. present
      2. absent
35. Stapes: width of crura relative to size of foramen; O’Leary & Gatesy 2008 c61
    - States
      1. narrower than stapedial foramen width
      2. Wider than stapedial foramen width
36. Bony Labyrinth: Posterior Entry of Lateral Semicircular Canal; Ekdale, 2013 c1
    - States
      1. Secondary Common Crus
      2. Posterior Ampulla
      3. 2= Vestibule
37. Bony Labyrinth: Largest Semicircular Arc Radius of Curvature; Ekdale, 2013 c3
    - States
      1. Anterior Semicircular Canal
      2. Lateral Semicircular Canal
      3. 2= Posterior Semicircular Canal
38. Bony Labyrinth: Position of the Lateral Semicircular Canal with respect to Posterior Semicircular Canal; Ekdale, 2013 c2
    - States
      1. low
      2. high
39. Bony Labyrinth: Shape of cochlear spiral; Ekdale, 2013 c4
    - States
      1. low
      2. high (>0.55)
40. Bony Labyrinth: Coiling of the cochlea; Ekdale, 2013 c5
    - States
      1. 1-2 cochlear turns
      2. 2-3 cochlear turns
      3. 3=over 3 turns
41. Bony Labyrinth: Percent volume of the cochlea; Ekdale, 2013 c6
    - States
      1. <50%
      2. 51-75%
      3. 2=>75%
42. Petrosal: Caudal tympanic process extension; Mateus, 2018 c2
    - States
      1. short (not exceeding the posterior process of the crista parotica)
      2. long (exceeds the posterior process of the crista parotica)
43. Petrosal: Caudal tympanic process position; Mateus, 2018 c3
    - States
      1. ventral to fenestra cochleae
      2. dorsal to fenestra cochleae
44. Petrosal: Hiatus Fallopii position; Mateus, 2018 c4
    - States
      1. terminally (anterior edge of the petrosal)
      2. ventral Side (tympanic)
45. Petrosal: Promontorial surface; Mateus, 2018 c6
    - States
      1. smooth
      2. not smooth
46. Petrosal: Subarcuate fossa size; Mateus, 2018 c8
    - States
      1. small
      2. wide
47. Petrosal: Epitympanic recess; Mateus, 2018 c9
    - States
      1. large
      2. small
48. Petrosal: Secondary facial foramen; Mateus, 2018 c10
    - States
      1. lateral to fenestra vestibuli
      2. anterior to fenestra vestibuli
49. Petrosal: Cochlear aqueduct position; Mateus, 2018 c11
    - States
      1. ventromedial face
      2. ventral face
50. Petrosal: Cochlear aqueduct slit; Mateus, 2018 c12
    - States
      1. present
      2. absent
